# The spin-forbidden transition in iron(IV)-oxo catalysts relevant to two-state reactivity

**DOI:** 10.1126/sciadv.ado1603

**Published:** 2024-06-28

**Authors:** Derek B. Rice, Deniz Wong, Thomas Weyhermüller, Frank Neese, Serena DeBeer

**Affiliations:** ^1^ Max Planck Institute for Chemical Energy Conversion, D-45470 Mülheim an der Ruhr, Germany.; ^2^ Helmholtz-Zentrum Berlin für Materialien und Energie, Hahn-Meitner-Platz 1, D-14109 Berlin, Germany.; ^3^ Max-Planck-Institut für Kohlenforschung, D-45470 Mülheim an der Ruhr, Germany.

## Abstract

Quintet oxoiron(IV) intermediates are often invoked in nonheme iron enzymes capable of performing selective oxidation, while most well-characterized synthetic model oxoiron(IV) complexes have a triplet ground state. These differing spin states lead to the proposal of a two-state reactivity model, where the complexes cross from the triplet to an excited quintet state. However, the energy of this quintet state has never been measured experimentally. Here, magnetic circular dichroism is used to assign the singlet and triplet excited states in a series of triplet oxoiron(IV) complexes. These transition energies are used to determine the energies of the quintet state via constrained fitting of 2p3d resonant inelastic x-ray scattering. This allowed for a direct correlation between the quintet energies and substrate C─H oxidation rates.

## Teaser

Resonant inelastic x-ray scattering reveals the triplet to quintet transition in iron(IV)-oxo complexes.

## INTRODUCTION

**Fig. 1.**
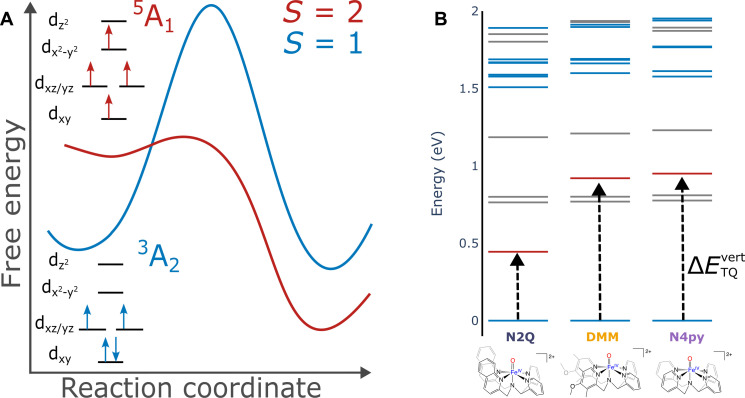
TSR model for iron(IV)-oxo complexes and calculated low-lying states for selected complexes. (**A**) Qualitative potential energy surface showing the proposed TSR model. (**B**) CASSCF-NEVPT2 calculated energies for low-lying singlet (grey), triplet (blue), and quintet (red) states. Arrows are highlighting the lowest vertical triplet to quintet excitation. Structures for the complexes are shown below.

Metal-centered spin-forbidden reactions play a vital role in chemical and biological reactions (*1*, *2*). A prominent example in biology is O_2_ binding to hemoglobin, where an *S* = 1 oxygen and *S* = 2 iron center bind to form an *S* = 0 product and in doing so must cross from these high spin reactant states to produce the low-spin product (*3*). Even seemingly, simple reactions, such as the reaction of the bare FeO^+^ cation insertion into H_2_, have been shown to proceed via multiple spin states (*1*, *4*). In chemistry, one system where it is less clear but that has been proposed to undergo spin-forbidden reactivity is nonheme *S* = 1 iron(IV)-oxo model complexes. In nature, enzymes such as TauD use a nonheme iron(IV)-oxo moiety with an S = 2 ground state that has been invoked for substrate oxidation (*5*). In contrast, the first synthetic nonheme iron(IV)-oxo model complex, and many of the model complexes to follow, have an *S* = 1 ground state (*6*, *7*). These *S* = 1 model complexes proved competent oxidants, although often not as powerful as their *S* = 2 counterparts (*7*–*10*). The theoretical analysis of these systems was pioneered by Shaik and coworkers and explained the reactivity of these model on the basis of two-state reactivity (TSR). In TSR, the S = 1 iron(IV)-oxo complex crosses to a relatively low-lying S = 2 state that facilitates reactivity via a reduced reaction barrier due to exchange-enhanced reactivity (Fig. 1A) (*11*–*17*). For hydrogen atom abstraction, calculations have predicted that the *S* = 1 iron(IV)-oxo complexes can reach four potential transition states, of which the *S* = 2 reaction pathway is the lowest in energy (*18*). Experimental explorations of this theory have been developed via synthetic means and using techniques such as infrared photodissociation spectroscopy (*19*, *20*). It has also been shown that the kinetic isotope effect could act as a way of elucidating which spin surface that the reaction proceeds on (*11*). When comparing the *S* = 1 iron(IV)-oxos in solution, reactivity experiments have established relationships between the theoretical triplet-quintet energy gaps (*21*, *22*). However, it must be clearly recognized that the theoretical prediction of accurate spin-state energetics in transition metal complexes remains a major challenge for quantum chemistry. Even the best correlated ab initio methods, such as those based on coupled-cluster theory, have great difficulties to overcome the enormous bias of the parent Hartree-Fock (HF) method in favor of high-spin states. In a less pronounced way, “pure” density functional theory (DFT) that does not incorporate HF exchange has the opposite bias. It is therefore not surprising that hybrid DFT to some extent cancels these two large errors. However, the cancelation is not highly systematic and, for example, does not carry over from one metal to the other or from one oxidation state to the next (*23*–*25*).

Despite this attention paid to the TSR both experimentally and theoretically, an experimental measurement of the triplet to quintet excitation energies has yet to be obtained. This is not due to a lack of spectroscopic investigations, as a multitude of techniques probing electronic excitations have been used to interrogate these complexes. In particular magnetic circular dichroism (MCD) has given insight into the electronic structure of these complexes via analysis of their spin-allowed transitions (*26*, *27*). The work by Decker *et al.* (*26*, *28*) correlates the MCD spin-allowed triplet-to-triplet excitations for a series Fe(IV)-oxo complexes to the ground state d-orbital splittings and uses this to make indirect correlations to reactivity. In two separate related studies, Kupper *et al.* (*27*) and Ye *et al.* (*29*) showed that a tetracarbene-ligated Fe(IV)-oxo complex caused a notable change in the ligand field splitting, as determined by observed spin allowed transitions in the MCD. These experimental data were then correlated to calculations, which showed that the tetracarbene complex should have an unusually large triplet to quintet splitting due to the strong in-plane donation of the carbene. However, the triplet to quintet transitions, which are correlated to reactivity, have never been observed experimentally. This may be attributed to the predicted low energy, often below the limit of conventional optical spectrometers (≤5000 cm^−1^) and the spin-forbidden nature of the transition, leading to weak spectroscopic intensities.

A technique that has shown great promise in measuring low-lying excited states is 2p3d resonant inelastic x-ray scattering (RIXS). 2p3d RIXS is a photon-in photon-out spectroscopy that uses an excitation energy tuned to an L-edge absorption resonance. The initial iron 2p to 3d L-edge excitation (~708 eV for iron) leads to an intermediate 2p^5^3d^n+1^ state, and then subsequent emission of a photon leads to a dipole-allowed decay to a final 2p^6^3d^n’^ state, which may be identical to the initial state (elastic scattering) or an excited d-d or charge-transfer (CT) state (inelastic scattering). The difference in energy between the final and initial state is called the energy transfer, which will be equal to optical transition energy. Because of the metal 2p orbital’s large spin-orbit coupling, spin-forbidden transitions become allowed. In recent years, 2p3d RIXS has seen increasing use in the fields of solid state physics and heterogenous catalysts (*30*–*33*) and, most recently, for limited applications to molecular complexes (*34*–*38*).

Here, we use a combination of 2p3d RIXS together with MCD to probe the energetics of the spin-allowed and spin-forbidden d-d transitions in a series of Fe(IV)-oxo complexes in order to obtain, to our knowledge, a direct experimental measure of the triplet to quintet gaps that are essential for direct correlation with TSR models. Three complexes were studied that feature a pentadentate N5 ligand, **N2Q**, **DMM**, and **N4py** (*21*, *39*, *40*).

## RESULTS

### Magnetic circular dichroism

We begin by evaluating the variable-temperature, variable-field (VTVH) MCD data for this series of complexes (see the Supplementary Materials for full details). The MCD data have been obtained from 5200 to 28,000 cm^−1^. This thus allows us to experimentally evaluate a broad range of transitions and to incorporate more of the low-energy part of the valence excitation spectrum, which is essential for understanding reactivity.

**Fig. 2.**
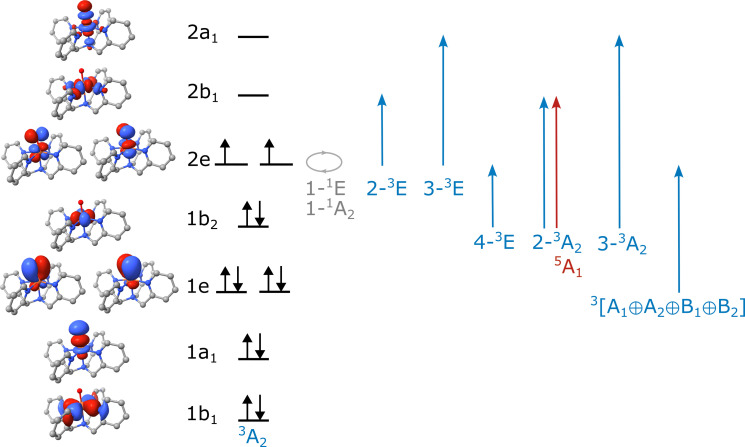
Overview over molecular orbitals and possible terms arising from single particle excitations relative to the ^3^A_2_ ground state of the target pseudo-*C*_4*v*_ complexes. Blue arrows and terms represent the spin- and dipole-allowed transitions. The gray arrow and terms represent the spin-forbidden triplet to singlet transitions within the 2e set of orbitals. The red arrow and term represent the lowest energy spin- and dipole-forbidden triplet to quintet. [The plots show CASSCF natural orbitals (excluding the second d-shell) that were used in the calculation].

To arrive at a reliable assignment of the MCD spectrum, complete active space self-consistent field (CASSCF)/N-electron valence perturbation theory (NEVPT2) calculations have been used as a guide for the spectral assignments. For the spin-allowed transitions, a standard (12,9) active space is used (Fig. 2), while for the low-lying quintet and singlet states, the double shell is used. Further information is available in Materials and Methods and shown in fig. S1. The molecular orbital scheme of Fe(IV)═O units in approximate *C*_4*v*_ symmetry is well known. The splitting of the molecular orbitals derived from the iron 3d set is dominated by the strong covalent bond formed between the iron and oxo units and is commonly in the order 1b_2_ (d_xy_, lone pair), 1e (d_xz,yz_, π-antibonding with O^2−^), 2b_1_(d_x2-y2_; σ-antibonding with the equatorial ligands), and 2a_1_ (d_z2_; σ-antibonding with O^2−^). The ^3^A_2_ ground state has the electronic configuration (1b_2_)^2^(2e)^2^. One electron excitations in this manifold give rise to a relatively complex series of multiplets. First, excitations within the 2e subshell give rise the two singlet states (1-^1^E and 1-^1^A_2_). These states are independent of the ligand field. Next, excitations out of the degenerate set of Π* orbitals into the antibonding 2b_1_(d_x2-y2_) and 1a_1_(d_z2_) give rise to two pairs of double degenerate triplet states (2-^3^E and 3-^3^E), while a fourth pair of degenerate states is formed by the excitations from the nonbonding 1b_2_ (d_xy_) orbital into the 1e(d_xz,yz_) pair. The first quintet state, ^5^A_1_, can be formed by a spin-flip excitation of the 1b_2_(d_xy_) → 2b_1_(d_x2-y2_). The energy of this state is primarily determined by the strength of the equatorial ligand field. For extremely strong equatorial ligands, it may even cross with 1-^5^B_1_(1b_2_(d_xy_) → 2a_1_(d_z2_)) (*27*).

**Fig. 3.**
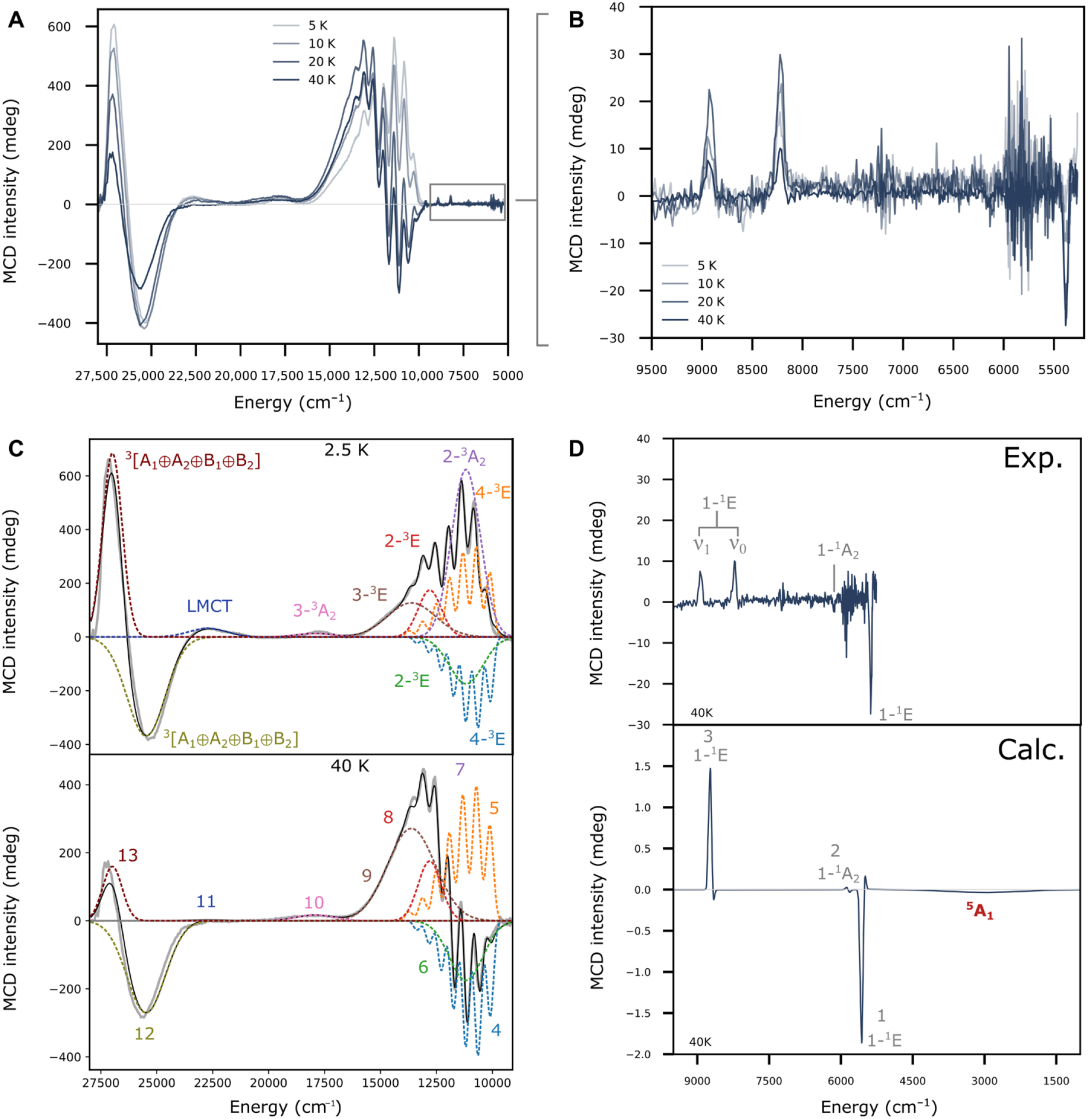
Magnetic circular dichroism spectra for N2Q. (**A**) Full variable temperature MCD spectrum for **N2Q** at 10 T. (**B**) Variable temperature MCD of **N2Q** at 10 T focusing on the low-energy spin-forbidden transitions. (**C**) Gaussian deconvolution of **N2Q** at 2.5 and 40 K and 10 T. (**D**) Experimental and calculated 40-K MCD spectrum over a range an expanded range of 1000 to 9500 cm^−1^. Calculated spectrum uses state-averaged CASSCF-NEVPT2 with linewidths of 75 cm^−1^ for the singlet transitions and 1800 cm^−1^ for the quintet transition. Experimental region below 5000 cm^−1^ is outside the detection limit for the MCD. Excited states are labeled with their corresponding label from Fig. 2. Numerical labels are for reference in table S4 and Fig. 4.

The discussion of the MCD for the **N2Q** data can be broken into two sections, the ultraviolet/visible (UV/Vis) region (>10,000 cm^−1^; Fig. 1A) and the near-infrared (NIR) region (<10,000 cm^−1^; Fig. 1B). Further analysis of this complex as well as **DMM** and **N4py** are in the Supplementary Materials. For the UV/Vis region, the first prominent feature in the experimental MCD spectra is a pseudo-A term with vibronic progression observed around 12,500 cm^−1^. This region is rich with overlapping bands of varying temperature dependence, as seen in the large shift from positive to negative MCD intensity below 12500 cm^−1^ and a more moderate shift above 12,500 cm^−1^. On the basis of the observed polarizations (table S4) deduced from Gaussian deconvolution (Fig. 3C) of the temperature dependent data, this feature contains bands assigned to excitations to the excited states 4-^3^E(1b_2_(d_xy_) → 2e(d_xz,yz_)) (10,090 and 10,120 cm^−1^ for the 0-0 peaks), 2-^3^A_2_(1b_2_(d_xy_) → 2b_1_(d_x2-y2_)) (11,180 cm^−1^), and 2-^3^E(1e(d_xz,yz_) → 2b_1_(d_x2-y2_)) (11,150 and 12,860 cm^−1^). These bands are similar in energies to the previously assigned values to **N4py** (*26*). The vibronic progressions for the 4-^3^E band arises from elongation of the Fe═O bond in the excited states and has a vibrational splitting of 572 ± 28 cm^−1^ with a Huang-Rhys factor of 2.8, all consistent with literature data and assignments (*26*). Additional fits within this complex feature reveals an additional band that is assigned to the ^3^A_2_ → 3-^3^E(1e(d_xz,yz_) → 2a_1_(d_z2_)) (14,170 cm^−1^) transition. At 17,900 cm^−1^ is the isolated 3-^3^A_2_(1b_2_(d_xy_) → 2a_1_(d_z2_)). Last, at higher energies, we assign the intense pseudo-A term feature to the oxo-to-iron ligand-to-metal charge-transfer (LMCT) transition of π-symmetry [1e(π) → 2e(π*); bands observed at 22,830 and 25,700 cm^−1^]. They will be intense in both absorption and MCD due to the large-transition dipole moment inherent in transitions between bonding/antibonding orbital pairs with high covalency between the donor and acceptor orbitals. Last, there is a weaker feature at 22,440 cm^−1^ that we cannot assign with certainty. It probably arises from the transition of either a lone-pair orbital or an equatorial ligand-based orbital into the 2e(π*) orbitals. These transitions would not show up in our calculations because we are not able to include the relevant donor orbitals into the active space due to computational limitations.

For the NIR region of **N2Q**, our CASSCF/NEVPT2 calculations predict the 1-^1^E state to be split into two nondegenerate states, with the lowest-energy state at 5600 cm^−1^ and the higher-energy state at 8700 cm^−1^ (table S3). The higher-energy state shows a larger admixture of symmetry allowed 1e(π) → 2e(π*) LMCT states (~12% versus ~6%; table S1). The 1-1A_2_ state is predicted around 5800 cm^−1^. As expected, the energies of these transitions are nearly independent of the ligand framework since these are intra-orbital transitions (see Fig. 1B). We assign the sharp features in the MCD spectra observed at 5380 cm^−1^ to the lowest-energy state of the split 1-^1^E set (Fig. 3, B and D). At 6160 cm^−1^ is a very weak feature nearly within the baseline signal to noise; however, this feature is present in all complexes and shifts to a similar degree as all other NIR features. On the basis of the energy and intensity (Fig. 3D), this feature is assigned as the 1-^1^A_2_. These transitions are sharp because an intra-orbital re-arrangement does not lead to a considerable change in the bonding (*41*). Consequently, excited state distortions are small, and no substantial vibronic progression is triggered. The calculated (vertical) transition energies and MCD signs are in excellent agreement with experiment (table S3). At higher energies, there is a pair of sharp features centered at 8230 and 8945 cm^−1^. Because of the increased LMCT contribution to this state, there is a slight elongation in the Fe═O bond distance that causes this small vibronic progression. The splitting gives an excited state vibrational splitting of 715 cm^−1^, lower than the ground state value of 833 cm^−1^ but considerably larger than the above 572 cm^−1^ for the 4-^3^E bands (*21*).

The remaining question is whether the MCD data show evidence of the important ^3^A_2_ → ^5^A_1_(1b_2_ → 2b_1_(d_x2-y2_)) excitation. For **N2Q**, the CASSCF/NEVPT2 calculations predict the ^5^A_1_ state lower in energy than the spin-allowed transitions (Fig. 1B). In our calculations, these states are predicted to be 2500 cm^−1^ (**N2Q**), 7340 cm^−1^ (**DMM**), and 7660 cm^−1^ (**N4py**) with a pronounced dependence on the equatorial ligand field. Unlike the intra-orbital 1-^1^E and 1-1A_2_ excitations that are sharp owing to the lack of a vibronic progression, the ^3^A_2_ → ^5^A_1_(1b_2_ → 2b_1_(d_x2-y2_)) excitation is expected to trigger a substantial vibronic progression along the Fe-N_eq_ stretching coordinate because the 2b_1_(d_x2-y2_) orbital is strongly σ-antibonding between the iron and the equatorial nitrogens. Thus, we expect a rather broad and very weak feature given the spin-forbidden nature of this excitation. We have attempted to calculate the band shapes of the 1-^1^E and 1-1A_2_ versus ^5^A_1_ using a DFT-based path integral formalism (*42*). The results indicate that the effective bandwidth of the singlets is expected to be around 150 cm^−1^, while the ^3^A_2_ → ^5^A_1_ band is expected to be around 3000 cm^−1^ wide. The experimental bandwidth of the singlet is 72 cm^−1^, demonstrating qualitative but not quantitative agreement between theory and experiment. If the calculated ratios are reasonable, then we may expect ^3^A_2_ → ^5^A_1_ to be in the range of 1500 to 2000 cm^−1^ wide. This is in range of the similar spin-allowed ^3^A_2_ → 2-^3^A_2_(1b_2_ → 2b_1_(d_x2-y2_)) transition found experimentally, which had full width at half maximum (FWHM) ranging from 1500 to 2300 cm^−1^ (table S4). Using values of 1800 cm^−1^ for the ^3^A_2_ → ^5^A_1_ and 75 cm^−1^ for ^3^A_2_ → 1-^1^E and 1-1A_2_, together with the calculated MCD intensities from CASSCF/NEVPT2, result in the calculated MCD spectra for **N2Q** shown in Fig. 3D. Here, the sharp peaks correspond to ^3^A_2_ → 1-^1^E and 1-1A_2_, and the very broad, very weak feature around 3200 cm^−1^ corresponds to ^3^A_2_ → ^5^A_1_. However, Note that transitions below 5000 cm^−1^ are outside the accessible range of our MCD spectrometer. It should also be mentioned that the exact energies of these transitions are exceedingly difficult to predict with quantitative accuracy. The reason for this is the enormous bias of self-consistent field methods (such as CASSCF) toward high-spin systems, which lead to very large differential dynamic correlation effects that are difficult to capture quantitatively with present day methodology. These limitations in theoretical approaches further underscore the need for experimental methods to observe these transitions, as will be described in the sections that follow.

Given the low sensitivity of optical spectrometers in this spectral region, the broad nature and intrinsically low intensity of the spin-forbidden ^3^A_2_ → ^5^A_1_ excitation, it is readily conceivable that it may not be possible to detect this important transition in conventional MCD spectrometers. However, we will argue below that the detailed assignment of the observable part of the MCD spectrum will put strong constraints on the assignment of the 2p3d RIXS spectra that allows us to confidently assign the ^5^A_1_ state and its energy relative to the ^3^A_2_ ground state.

### 2p3d RIXS

As the triplet to quintet transition is the main transition of interest for the interpretation of the reaction mechanism, a different method of probing d-d transitions is therefore necessary. Here, the 2p3d RIXS is measured by setting the incident energy and measuring the emission giving a slice of the two-dimensional plane at constant incident energy, and differences in the spectra are seen as a function of this incident energy. In the Supplementary Materials, there are RIXS cuts shown at four incident energies for the three iron(IV)-oxo complexes and the iron(II) precursors. The MCD spectra and selected RIXS cuts (at incident energies that best highlight the modulations in the low-energy region of the spectra) are shown for the three iron(IV)-oxo complexes in Fig. 3.

**Fig. 4.**
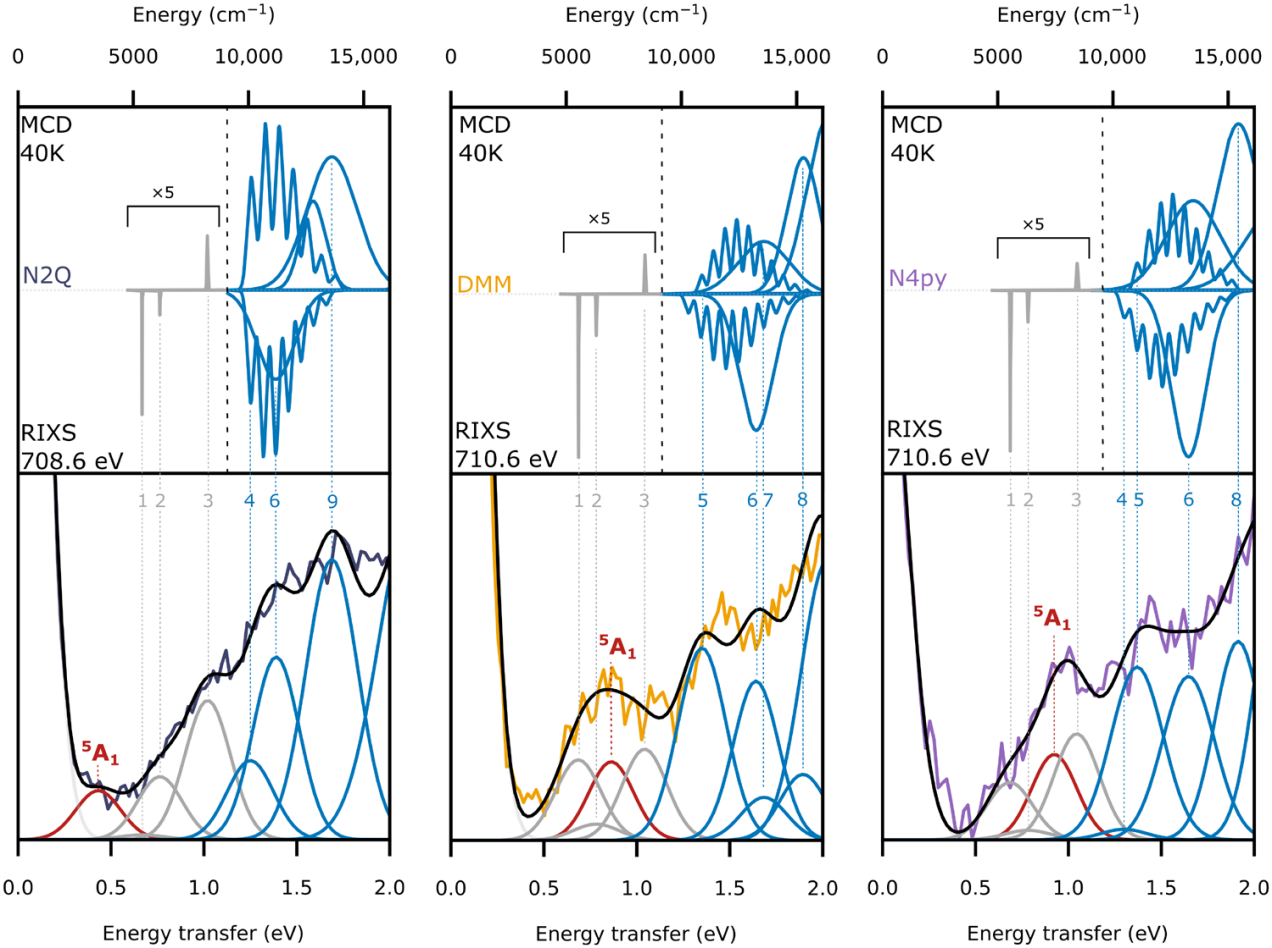
Comparison of RIXS cuts and MCD spectra. The 40-K, 10-T MCD fits to the MCD data (fits and spectra overlaid in Fig. 3 and fig. S5) (top), and 2p3d RIXS cuts at the indicated incident energy (bottom) for the three complexes. The fits of the spectra include the triplet transitions from MCD (blue), the singlet transitions from NIR-MCD (grey), and the freely fitted low-energy peak that is assigned as the quintet (red). The elastic line (light gray) is added for fit completion. Peak labels numbers correspond to the MCD peaks above and in table S4.

The displayed RIXS cuts show a similar trend in all three complexes, with intense maxima at 2.5 eV (~20,000 cm^−1^), a shoulder at ~1.5 eV (~12,100 cm^−1^), and then either a shoulder or peak below 1.0 eV (~8065 cm^−1^). Because the energy transfer range for 2p3d RIXS is analogous to UV-Vis/NIR, the transitions determined from MCD can be used to fit the RIXS to determine the transitions contributing to the spectra. We note, however, that the RIXS intensity mechanism is different than that of MCD, and 2p spin orbit coupling in the intermediate state can provide an mechanism for formally spin forbidden transitions to gain intensity (*31*, *43*). Figure 4 shows fits to selected energy transfer cuts of the 2p3d RIXS with the energies of the Gaussians fixed to the MCD values for the triplet (blue Gaussians) and singlet transitions (gray Gaussians). Between 0.4 (~3230 cm^−1^) and 1.0 eV, there is also intensity in all three complexes, which cannot be accounted for by just the singlet transitions. This is particularly noticeable in the **N4PY** and **N2Q** RIXS, where the additional intensity appears either above or below the lowest energy singlet, respectively. Addition of one function with a floating center is needed to properly model the low energy spectral region for all three complexes. The additional features, not observed in the MCD, are shown as a single red Gaussian in each of the RIXS fits. As the triplet to singlet transitions have been assigned via MCD, these additional features must correspond to the excited quintet final states. The energy of these transitions increases from 0.45 eV (**N2Q**) > 0.86 eV (**DMM**) > 0.94 eV (**N4py**), agreeing with the trend calculated in Fig. 1 of 0.31 eV (**N2Q**) > 0.91 eV (**DMM**) > 0.95 eV (**N4py**). These fits give evidence of an experimentally measured triplet to quintet transition in non-heme iron(IV)-oxo complexes.

## DISCUSSION

### Correlating quintet energies to C─H bond activation

**Fig. 5.**
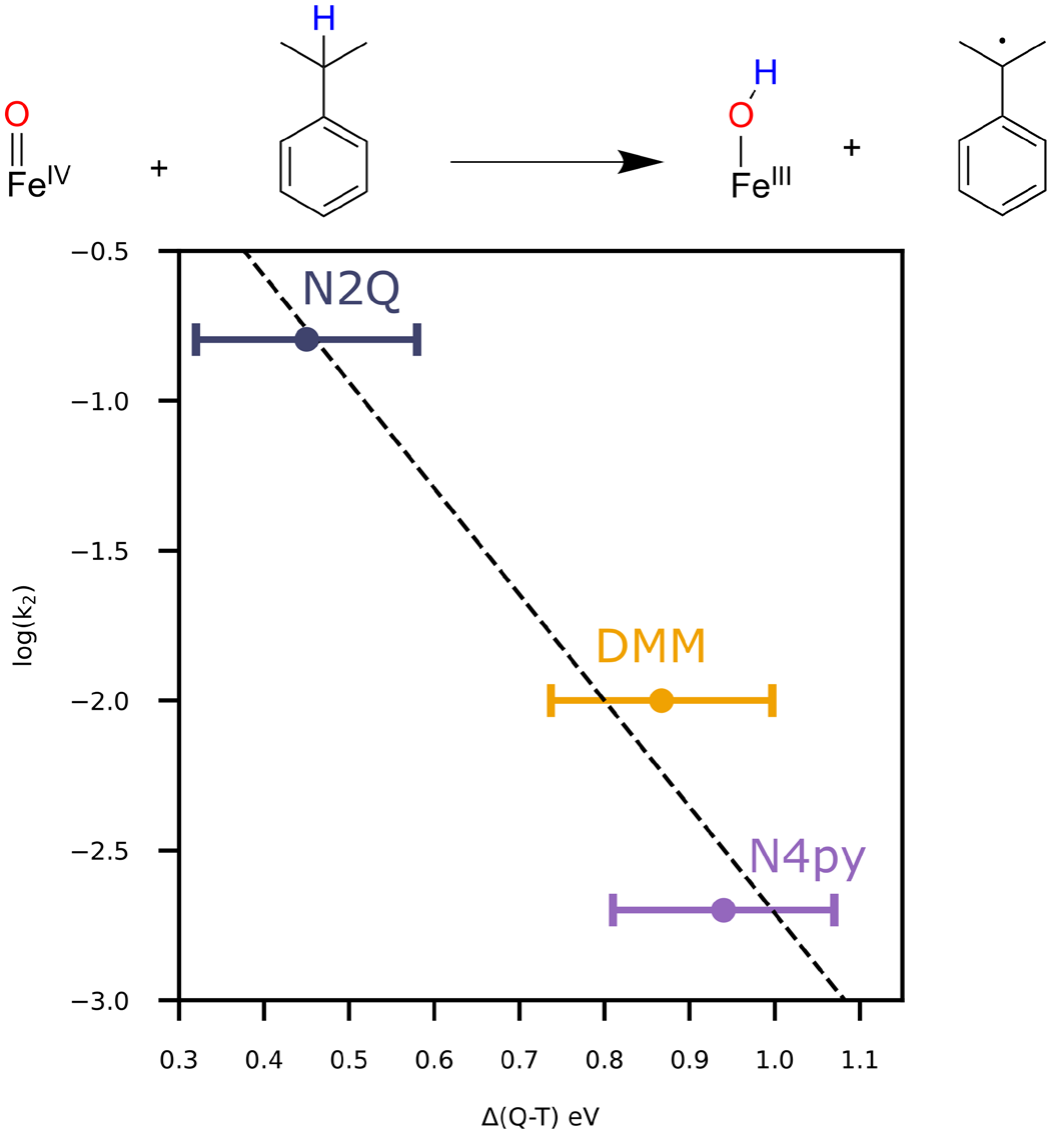
Plot of the log of the second-order rate constant for oxidation of cumene as a function of 2p3d RIXS determined ^5^A_1_ energy. Error bars are an upper limit based on the FWHM of the elastic line.

Having experimentally measured the triplet to quintet transition, we have the unique opportunity to directly correlate the relative energetics determined from our spectroscopic studies to the experimentally determined rate constants for C─H oxidation. The lower the energy of the ^5^A_1_, the easier it is to cross from the triplet surface to the quintet surface while the system is attacking C─H bonds. Consequently, one expects a clear correlation between the position of the ^5^A_1_ state and the second order rate constant for C─H bond activation. Note that the best comparison would be to the energetics of the equilibrium structure on the ^5^A_1_, but the reduction in energy due to geometric relaxation for these complexes are calculated to be similar in energy: 0.17, 0.21, and 0.24 eV for **N2Q**, **DMM**, and **N4py**, respectively. With a similar expected relaxation, the vertical transition energies can be used to correlate with the reactivity. For the three complexes presented here, second-order rate constants were previously determined with the substrate cumene (bond dissociation free energy of 84.5 kcal/mol) (*21*, *39*). The correlation between this data and our measurements is shown in Fig. 5, allowing an experiment-to-experiment relation to be made between spectroscopy and reactivity in these Fe(IV)-oxo systems. Previous studies all relied on the correlation of calculated triplet to quintet excitation energies to experiment (*15*, *22*). Given the enormous difficulty to calculate these energy differences accurately, it is important to establish these trends experimentally to properly probe the TSR model. Thus, these results provide direct experimental evidence to support the TSR model and demonstrate a viable method for measuring spin-forbidden transitions in model complexes.

In summary, using a combination of MCD and 2p3d RIXS, the low-energy spin-forbidden transitions in iron(IV)-oxo complexes were experimentally measured. MCD provided high-resolution determination of the triplet to singlet transitions between 5000 and 10,000 cm^−1^. 2p3d RIXS was then used to assign the last remaining transition below 10000 cm^−1^, the triplet to quintet. The quintet state for this transition has long been postulated as relevant to the reactivity of iron(IV)-oxo complexes via the TSR mechanism. Using this transition energy, the experimental correlation between the triplet to quintet energies and the rate constants was finally possible. These studies can be extended beyond the complexes presented here to further interrogate the generalizability of the TSR model in catalysis research. The combined MCD and 2p3d RIXS approach presented here provides a direct experimental probe of spin state energetics, which is vital for assessing theoretical methods, where experimental validation for spin-forbidden energetics has been severely lacking. This work demonstrates that the 2p3d RIXS technique can be applied to other systems, where the experimental knowledge of the spin-state energetic is essential to understanding function. This not only applies in catalysis research but also has broad implications in all areas of chemistry and materials research, where detailed knowledge of the valence excitation spectrum can ultimately guide rational design.

## MATERIALS AND METHODS

### Synthesis and sample preparation

The ligand for the N4py derivatives N4py, DMM, and 2PyN2Q was all synthesized as previously described (*21*, *39*, *44*, *45*). The iron(II) salts were generated in a nitrogen-filled glovebox by mixing 1 equivalent of Fe(II)(OTf)_2_(MeCN)_2_ in dried/distilled MeCN with the desired ligand and allowing to stir for 1 hour. Recrystallizations were performed via vapor diffusion using diethyl ether into MeCN. The respective iron(IV)-oxo complexes were generated as describe previously for **N4py** and **N2Q**, where the iron(II) was dissolved in minimal MeCN outside of the box, and 2 equivalents of cerium ammonium nitrate dissolved in ultrapure H_2_O is added, and the mixture was allowed to stir for approximately 1 min. Approximately 20 equivalents of NaPF_6_ were then added, and the solution was immediately cooled in an ice bath when participate begins to form immediately. After 10 min of cooling, the mixture was filtered and rinsed with cool H_2_O. The solid was further dried and stored in a −80°C freezer until further use. For the x-ray diffraction (XRD) of **DMM**, in place of adding NaPF_6_, NaClO_4_ (20 equivalents) was added and the procedure was identical after. After solid was isolated, recrystallizations were set up by dissolving the complex in minimal MeCN and layering on top of an aqueous NaClO_4_ solution. Slow evaporation formed dark blue crystals suitable for XRD.

For 2p3d RIXS, powder samples were collected for the iron(II) and iron(IV)-oxo of **N4py**, **DMM**, and **N2Q**. For the iron(IV)-oxo samples, the PF_6_ salts were used, and for the iron(II) samples, the OTf salts were used. The samples were spread as powders onto double-sided carbon tape that was affixed to a copper sample holder.

For MCD, samples were prepared in a nitrogen-filled glovebox. The powder was dissolved in a 75:25 PrCN:MeCN solvent mixture precooled to −40°C into precooled MCD cells and quickly brought out and immediately frozen in liquid nitrogen. For the mull sample, solid **N2Q** was mixed with pre-cooled mulling agent using a pre-cooled mortar and pestle.

### X-ray crystal structure determinations

Blue prismatic crystals of **DMM** (CCDC 2314930) were covered with polyfluorinated polyether and selected under a microscope with an applied nitrogen cryo-stream at about −40°C in polarized light. A suitable crystal was picked up with a nylon loop and rapidly mounted in the nitrogen cold gas stream of the diffractometer at 100 K. A Bruker Kappa Mach3 APEX-II diffractometer with a Bruker IμS x-ray source and INCOATEC Helios mirror optics (Mo-Kα radiation; λ = 0.71073 Å) was used. Final cell constants were obtained from least squares fits of setting angles of several thousand strong reflections. Intensity data were corrected for absorption using intensities of redundant reflections using SADABS (*46*). The structure was readily solved by direct methods and subsequent difference Fourier techniques. The Bruker APEX3 (*47*) software package was used for solution and refinement of the structures. All non-hydrogen atoms were anisotropically refined, and hydrogen atoms were placed at calculated positions and refined as riding atoms with isotropic displacement parameters. Crystal and data collection details are given in tables S7.

### RIXS measurements

All measurements were performed at the PEAXIS beamline of BESSY II (*48*). The scattering angle was fixed to 60°. The incident beam spot size was 4 (*h*) × 40 (*v*) μm. The resolution was estimated to be ~270 meV from the FWHM of the elastic line. During the energy transfer scans (at fixed incident energies), the samples were moved continuously in a meandering pattern at a horizontal rate of 100 μm/s and vertical rate of 1000 μm/s to avoid sample damage. Scans below 50 μm/s showed notable shifts in the RIXS cuts, demonstrating that the maximum effective residence time per spot is 0.3 s. Because of the rapid sample damage, undamaged x-ray absorption spectroscopy spectra could not be obtained, as scanning the energy of incident beam adds a considerable overhead in time per spot, which is above the allowable dose. The inhomogeneity of the powder sample prevents absorption scans while continuously moving. Incident energies are referenced to the Fe_2_O_3_ L_3_ maxima of 708.5 eV.

Fitting of the iron(IV) cuts was carried out by using a Gaussian deconvolution. When using MCD fitted data, energies of individual functions were set to corresponding energies determined by the MCD. The FWHM was allowed to float from a minimum of the elastic line FWHM up to a maximum of the MCD determined FWHM. A freely floating peak was used to fit the additional intensity below 1 eV. For comparison, fitting was also performed using freely floating Gaussian functions, where the FWHM of the Gaussian functions was set to the value determined by the elastic line FWHM.

### MCD measurements

All measurements used an Olis DSM17 CD spectropolarimeter with samples placed in an Oxford cryostat Spectromag SM4000. Temperature ranges were from 2 to 40 K. Energies from 5000 to 10,000 cm^−1^ were collected using an InGaAs detector and an accumulation time of 4 s. From 9000 to 30,000 cm^−1^, the spectra were collected using a Hamamatsu R316 PMT with accumulation time of 1 s. For the low-energy NIR features, the final spectra were the averages of four scans due to the low signal-to-noise ratio.

The positions at which VTand VTVH MCD data were taken were determined from the results of the Gaussian fit resolution of the absorption and MCD spectra. Gaussian peak energies and bandwidths were held constant throughout all temperatures and fields, and intensities were allowed to float unconstrained except for pseudo-A terms that were constraint to opposite signs. NIR baseline was corrected using a second-order polynomial fit to each individual spectrum. Because of the temperature dependence and weak signal, VTVH for the NIR features below 5 K are nearly indistinguishable from the noise and not used in the fits. Spin Hamiltonian simulations were performed by fitting the MCD intensity as a function of μ*B*/*kT*, where μ is the Bohr magneton, *k* is the Boltzmann constant, *B* the strength of magnetic induction, and *T* is temperature in Kelvin.

### Mössbauer measurements

All samples were measured on unenriched solid powder samples (PF_6_ salts) with a conventional Mössbauer spectrometer with constant acceleration of γ-source (^57^Co/Rh, 18 GBq, kept at room temperature). The minimum experimental linewidth was 0.27 mm/s. The sample temperature was maintained using an Oxford Instruments Variox cryostat. Isomer shifts are relative to iron metal foil.

### Calculations

All calculations were performed using the ORCA program package version 5.0.3 (*49*). For CASSCF/second-order NEVPT2 or CASSCF/NEVPT2 calculations (*50*–*52*), geometries were taken from the crystal structures, and hydrogen atoms were optimized using the BP86 functional (*53*). The def2-TZVP basis set was used for iron and the first coordination sphere, while the def2-SVP basis was used for all hydrogens and carbons (*54*). The resolution of identity (RI) approximation was used with the “AutoAux” keyword in ORCA (*55*). For the orca_esd calculations, the XRD structures were optimized fully to the lowest triplet, quintet, and singlet geometries, and time-dependent DFT (TD-DFT) was used to optimize the two higher-energy singlet structures. For the full optimizations, the conductor-like polarizable continuum continuum solvation model was used with the preset values for acetonitrile.

For state specific CASSCF/NEVPT2 calculations of the ^3^A_2_, ^5^A_1_, ^1^E, and ^1^A_2_ states, an active space of twelve electrons in fifteen orbitals (12,15) was used for the triplet state, while an active space of (12,16) was used for the quintet state. The active space orbitals consist of the 3d orbitals (3d_xy_, 3d_xz,_, 3d_yz,_ 3d_x2-y2_, and 3d_z2_), the bonding oxygen-based orbitals (2p_x_, 2p_y_, and 2p_z_), the equatorial bonding orbital (N 2p). In addition, the active space was further improved to describe the important Fe═O bond more accurately. To this end, three additional empty iron-centered d orbitals (4d_xy_, 4d_xz_, and 4d_yz_) were included in the active space together with a second p-shell on the oxo-oxygen (3p_x_, 3p_y_, and 3p_z_). Because the quintet state occupies an additional d orbital, an additional iron “4d” orbital (4d_x2-y2_) was included in the active space for these calculations. For MCD simulations and the higher-energy states, a (12,9) active space, excluding the doubleshell, was used with state averaging of 5 quintets, 45 triplets, and 25 singlets.

The orca_esd package (*42*) was used to estimate the vibrational broadening due to geometric distortions in the excited state. The ^3^A_2_, ^5^A_1_, and ^1^A_2_ structures were obtained from DFT optimizations on the respective spin surface from the crystal geometry. The ^1^E were not the lowest-energy DFT structures and were obtained from TD-DFT optimizations targeting the specific roots. The respective Hessians were then used for inputs into the excited-state dynamics (ESD) block to calculate absorption spectra. The values obtained from orca_esd were ~150 and 3000 cm^−1^ for the singlets and quintet, respectively. This is an overestimate of the experimental ~75 cm^−1^ for the singlets. For the quintet, as no direct value has been obtained, a comparison of the value to the ^3^A_2_ → 2-^3^A_2_ transition that also populates the equatorial antibonding orbital, although on the same spin surface, has values ranging from 1500 to 2400 cm^−1^.

## Supplementary Materials

### This PDF file includes:

Figs. S1 to S15 Tables S1 to S9 References
