# Supplementary material for: The spin-forbidden transition in iron(IV)-oxo catalysts relevant to two-state reactivity
Source: Sci Adv. 2024 Jun 28;10(26):eado1603. doi: 10.1126/sciadv.ado1603 (PMC11212722; doi:10.1126/sciadv.ado1603)
Supplement: Supplementary file 1 — Figs. S1 to S15 Tables S1 to S9 References [file sciadv.ado1603_sm.pdf]

Science Advances
AAAS

Supplementary Materials for

The spin-forbidden transition in iron(IV)-oxo catalysts relevant to two-state reactivity

Derek B. Rice *et al.*

Corresponding author: Serena DeBeer, serena.debeer@cec.mpg.de; Frank Neese, neese@kofo.mpg.de; Derek B. Rice, derek.rice@cec.mpg.de

Sci. Adv. **10**, eado1603 (2024)
DOI: 10.1126/sciadv.ado1603

This PDF file includes:

Figs. S1 to S15
Tables S1 to S9
References

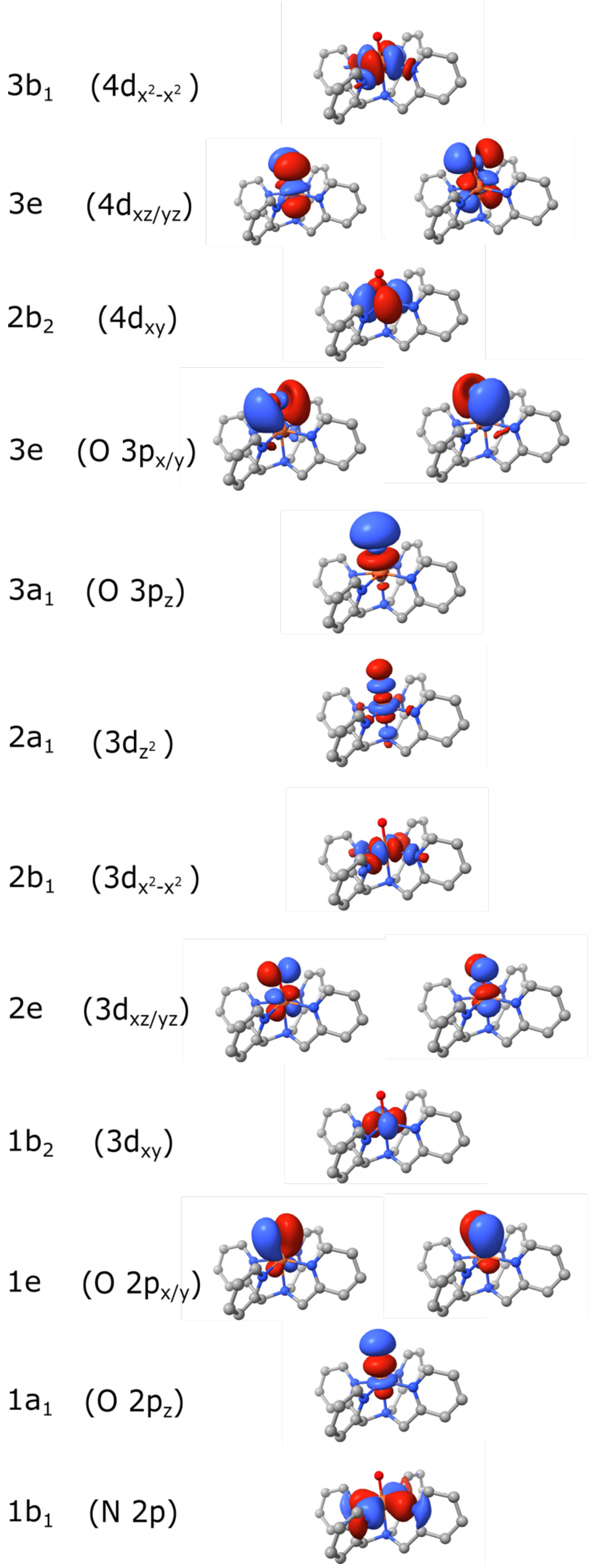

Figure S1. Active space orbitals used in the state-specific calculations.

Table S1. CASSCF configurations and weights for state specific triplet and quintet states and the three state averaged singlet states. Doubleshell orbitals are excluded from table and all are unoccupied.

Complex	Spin	Weight	σ_{eq}	σ_z	π_x	π_y	xy	π^*_x	π^*_y	σ^*_{eq}	σ^*_z
N4py	S = 1 ($^3A_2$)	0.825	2	2	2	2	2	1	1	0	0
		0.028	2	2	1	1	2	2	2	0	0
		0.024	2	1	1	2	2	2	1	0	1
		0.023	2	1	2	1	2	1	2	0	1
	S = 2 ($^5A_1$)	0.814	2	2	2	2	1	1	1	1	0
		0.032	2	1	2	2	1	1	1	1	1
		0.03	2	1	1	2	1	2	1	1	1
		0.026	2	1	2	1	1	1	2	1	1
		0.025	2	0	2	2	1	1	1	1	2
	S = 0 (^1E)	0.433	2	2	2	2	2	2	0	0	0
		0.359	2	2	2	2	2	0	2	0	0
		0.427	2	2	2	0	2	2	2	0	0
		0.343	2	2	2	2	0	2	2	0	0
	S = 0 ($^1A_2$)	0.793	2	2	2	2	2	1	1	0	0
		0.077	2	2	2	1	1	2	2	0	0
		0.016	2	2	1	2	1	2	1	1	0
		0.016	2	2	1	1	2	1	2	1	0
	S = 0 (^1E)	0.412	2	2	2	2	2	0	2	0	0
		0.338	2	2	2	2	2	2	0	0	0
		0.061	2	2	2	2	0	2	2	0	0
		0.051	2	2	2	0	2	2	2	0	0

			σ_{eq}	σ_z	π_x	π_y	x_z	π^*_x	π^*_y	σ^*_{eq}	σ^*_z
DMM	S = 1 ($^3A_2$)	0.82	2	2	2	2	2	1	1	0	0
		0.029	2	2	1	1	2	2	2	0	0
		0.024	2	1	1	2	2	2	1	0	1
		0.024	2	1	2	1	2	1	2	0	1
	S = 2 ($^5A_1$)	0.808	2	2	2	2	1	1	1	1	0
		0.033	2	1	2	2	1	1	1	1	1
		0.029	2	1	1	2	1	2	1	1	1
		0.028	2	1	2	1	1	1	2	1	1
		0.024	2	0	2	2	1	1	1	1	2
	S = 0 (^1E)	0.375	2	2	2	2	2	2	0	0	0
		0.345	2	2	2	2	2	0	2	0	0
		0.065	2	2	2	2	2	1	1	0	0
		0.042	2	2	2	0	2	2	2	0	0
		0.039	2	2	2	2	0	2	2	0	0
	S = 0 ($^1A_2$)	0.721	2	2	2	2	2	1	1	0	0
		0.081	2	2	2	1	1	2	2	0	0
		0.035	2	2	2	2	2	2	0	0	0
		0.031	2	2	2	2	2	0	2	0	0
		0.016	2	2	1	2	1	2	1	1	0
		0.015	2	2	1	1	2	1	2	1	0
	S = 0 (^1E)	0.388	2	2	2	2	2	0	2	0	0
		0.354	2	2	2	2	2	2	0	0	0
		0.062	2	2	2	2	0	2	2	0	0
		0.057	2	2	2	0	2	2	2	0	0

			σ_{eq}	σ_z	π_x	π_y	x_z	π^*_x	π^*_y	σ^*_{eq}	σ^*_z
N2Q	S = 1 ($^3A_2$)	0.808	2	2	2	2	2	1	1	0	0
		0.027	2	2	1	1	2	2	2	0	0
		0.026	2	2	2	1	1	2	1	0	1
		0.025	2	2	1	2	1	1	2	0	1
	S = 2 ($^5A_1$)	0.791	2	2	2	2	1	1	1	1	0
		0.044	2	2	2	1	1	1	1	1	1
		0.032	2	2	1	1	2	1	1	1	1
		0.031	2	2	2	0	1	1	1	1	2
	S = 0 (^1E)	0.412	2	2	2	2	2	0	2	0	0
		0.351	2	2	2	2	2	2	0	0	0
		0.033	2	2	0	2	2	2	2	0	0
		0.031	2	2	2	0	2	2	2	0	0
	S = 0 ($^1A_2$)	0.764	2	2	2	2	2	1	1	0	0
		0.064	2	2	1	1	2	2	2	0	0
		0.017	2	2	1	1	2	1	2	0	1
		0.016	2	1	1	2	2	2	1	0	1
	S = 0 (^1E)	0.386	2	2	2	2	2	2	0	0	0
		0.328	2	2	2	2	2	0	2	0	0
		0.065	2	2	0	2	2	2	2	0	0
		0.053	2	2	2	0	2	2	2	0	0

Table S2. Vertical Triplet Quintet Energy Gaps (eV)

	B3LYP	State-Specific (12,9)	State Specific (12,16)
N4py	1.10	1.22	0.95
DMM	1.00	1.20	0.91
N2Q	0.47	0.68	0.31

Table S3. Experimental and CASSCF/NEVPT2 Triplet to Singlet Energy Differences (eV)

	**N2Q**		**DMM**		**N4py**	
	**Exp.**	**Theory**	**Exp.**	**Theory**	**Exp.**	**Theory**
^1E	0.667	0.764	0.686	0.793	0.687	0.783
$^1A_2$	0.762	0.800	0.781	0.828	0.782	0.820
^1E	1.018	1.211	1.043	1.262	1.047	1.250

MCD Band Assignment for Spin-Allowed Transitions.

The MCD spectra for all three complexes (Figures S2 and S3) show a complex temperature dependence below 16000 cm^{-1}. At lower temperatures the feature has nearly entirely positive MCD intensity, but as the temperature increases the low-energy portion of the spectra loses intensity until by 40K there is negative MCD intensity. The higher-energy portion of this region maintains a positive MCD, which increases with increasing temperature until ~20K then decreases in all complexes at higher temperatures. All three complexes show a resolved vibronic structure similar to what has been seen in previous MCD studies on iron(IV)-oxo complexes. A similar Franck-Condon analysis is applied to these bands in the section below. At higher energies for **N2Q** are weak, separated positive features followed by an intense negative and then positive derivative shape. For **DMM** and **N4py** the positive intensity is broader and relatively more intense, and there is only the strong negative feature at ~26000 cm^{-1}.

For interpretation of the MCD spectra, Gaussian deconvolution was used with minimal bands of 10 for **N2Q**, and 9 for **DMM** and **N4py**. Figure S4 shows the results of these fits at 2.5K and 40K for each of the three complexes. The energies and variable-temperature dependence (Figure S5) of the bands can be used along with NEVPT2 calculations to assign the states for each transition. For ligand-field excitations, NEVPT2 can get quantitatively accurate results (see Table S4). For charge-transfer (CT) transitions, or ligand-field transitions with considerable CT character, NEVPT2 can fail to accurately predict these energies if the ligand-based orbitals are outside of the active space.

Bands 4 and 5 are used to fit the vibronic structure described below, and together the two C-terms give a positive pseudo-A term (the higher energy of the two C-terms is positive). Despite the substantial overlap of the vibronic structure with additional bands, for **DMM** and **N4py** below 11000 cm^{-1} there is an isolated negative band at all temperatures, giving confidence to sign of the pseudo-A term by having lower-energy negative features. The temperature dependence of the bands give a largely xy dominated polarization, and taking this into combination with the sign of the pseudo-A term and vibronic progression (based on previous iron(IV)-oxo studies) would assign these bands as the $^3A_2 \rightarrow$ 4-$^3E(1b_2(d_{xy}) \rightarrow 2e(d_{xz/yz}))$ transitions. The NEVPT2 energies for the states support this assignment, as these are calculated to be the lowest energy spin-allowed transitions in all three complexes.

Bands 6, 7, 8 and 9 contain substantial overlap of intensity and lead to the complex temperature dependence of the spectra. Bands 6 and 8 are largely overlapping, which could lead to some uncertainty in the exact intensity of each band, however due to the eventual negative intensity at higher temperatures, both a positive and negative band are needed to accurately describe the spectra. Based on the agreement with calculated transitions, as well as the xy-polarization determined from temperature dependence, bands 6 and 7 are assigned as the two C-terms describing the positive pseudo-A term of

the $^3A_2 \rightarrow 2\text{-}^3E(2e(d_{xz/yz}) \rightarrow 2b_1(d_{x2-y2}))$ (*56*). With the three complexes present in the study, the absolute values can also be used along with the relative values between complexes for comparison of the experimental and calculated values. For the $^3A_2 \rightarrow 2\text{-}^3E$ transition, there is both an experimental and calculated shift of ~500 cm^{-1} **N2Q** to lower energy compared to **N4py**/**DMM**.

The temperature dependence of band 8 indicates it is a z-polarized transition. This has been previously assigned to the $^3A_2 \rightarrow 2\text{-}^3A_2$ $(1b_2(d_{xy}) \rightarrow 2b_1(d_{x2-y2}))$ or $^3A_2 \rightarrow 3\text{-}^3A_2$ $(1b_2(d_{xy}) \rightarrow 2a_1(d_{z2}))$ transitions in similar complexes, and calculated energies would support the assignment to the $^3A_2 \rightarrow 2\text{-}^3A_2$ $(1b_2(d_{xy}) \rightarrow 2b_1(d_{x2-y2}))$ transition. It has been predicted that the two-electron A_2 transition gains intensity due to the actual lower symmetry of the complex, C_s, which allows for mixing with nearby excited states. For the experimental values the transition in **N2Q** is ~2300 cm^{-1} lower than **N4py** and **DMM**, and the calculated values have **N2Q** 1400 cm^{-1} lower in energy, predicting a smaller difference but still larger than that seen for the $^3A_2 \rightarrow 2\text{-}^3E$ transition.

The temperature dependence of band 9 is nearly purely xy-polarized. This temperature dependence supports the assignment as the $^3A_2 \rightarrow 3\text{-}^3E$ $(2e(d_{xz/yx}) \rightarrow 2a_1(d_{z2}))$ transition. It would be expected for the transition to exhibit a pseudo-A term from the two C-term components, but these transitions are calculated to mix heavily with charge-transfer transitions, and intense CT transitions can distort the band shapes leading to a standard C-term feature (*56*). The $^3A_2 \rightarrow 3\text{-}^3E$ transition for **N2Q** is determined to be 3000 cm^{-1} lower in energy than for **N4py** and **DMM**. For **N4py** and **DMM**, the two states are calculated to be nearly degenerate while for **N2Q** the two states are predicted to be approximately 2000 cm^{-1} apart. Overall, the splitting between the states is 4100 cm^{-1} between the lowest energy **N2Q** 3-^{3}E state and **N4py**/**DMM**, and 1800 cm^{-1} between the higher energy **N2Q** 3-^{3}E state and **N4py**/**DMM**. This puts the average energy difference between the complexes at 3000 cm^{-1}, in excellent agreement with the experimental splitting.

For Band 10, the temperature dependence gives mixed polarization with considerable z-polarized character. This mixture of polarization leads to an assignment of the spin-allowed $^3A_2$ term, $3\text{-}^3A_2(1b_2(d_{xy}) \rightarrow 2a_1(d_{z2}))$. The 2-electron 3-^3A_2 term is calculated to have significant (>20%) LMCT mixing. For CASSCF/NEVPT2 calculations, there is an overestimation of excitation energies of CT excited states, leading to a larger deviation in the calculated energies vs experimental energies for these heavily mixed states. The values of the 3-^3A_2 deviate significantly from the experimental values, ~9000 cm^{-1} higher in energy. This has been seen consistently for the CASSCF/NEVPT2 calculations of this specific state.(*27*) The trend in relative energies, however, agrees very well with what is measured experimentally. **N2Q** is calculated to be 2000 cm^{-1} lower in energy than **N4py**/**DMM**, while experimentally it was determined to have a 3-^3A_2 state 2300 cm^{-1} lower in energy.

Finally bands 11, 12, and for **N2Q** band 13 reside at the upper limit of the energy range. These bands experimentally show small variation between the complexes, with bands 11 and 12 both ~400 cm^{-1} lower in energy for **N2Q** than **N4py**/**DMM**. This lack of ligand field effect could be indicative of LMCT states. In the CASSCF/NEVPT2 calculations the LMCT dominated states begin to arise above 30000 cm^{-1}. For $\pi \rightarrow \pi^*$ transitions, it would be expected that excitation from a highly covalent bonding orbital into the corresponding antibonding orbital would lead to an intense transition with significant MCD intensity. This would indicate band 12 (and 13) to arise from $\pi \rightarrow \pi^*$ transitions. Additionally, the excitation from the 1e to 2e would lead to the pseudo-A term as seen in the MCD. Finally, when comparing the $\pi \rightarrow \pi^*$ transitions between complexes, there is only a small shift in the calculated values of ~500 cm^{-1}, in agreement with the experimental shifts of band 12. For band 11, the small shift still suggests an LMCT, but the weak intensity would indicate excitation from less covalent, or non-oxygen ligand-based orbital. Including these orbitals into the active space would make the calculations too costly, and as such the exact assignment of band 11 cannot be obtained at this time.

Table S4. MCD Peaks and polarizations

N4py

band	energy (cm^{-1})	line width (cm^{-1})	dipole products Myz, Mxz, Mxy	%polarization (x,y,z)	energy calc. (cm^{-1})	assignment
4	10500	300	0.89, 0.89, 1.60	43.3, 43.3, 13.3	13090	4-^3E ($1b_2 \rightarrow 2e$) $d_{xy} \rightarrow d_{xz,yz}$
5	11060	300	0.89, 0.89, 1.60	43.3, 43.3, 13.3	13620	4-^3E ($1b_2 \rightarrow 2e$) $d_{xy} \rightarrow d_{xz,yz}$
6	13280	1358	0.83, 0.83, 1.92	45.7, 45.7, 8.6	13630	2-^3E ($2e \rightarrow 2b_1$) $d_{xz,yz} \rightarrow d_{x2-y2}$
7	13500	1895	1.25, 1.25, -0.36	7.1, 7.1, 85.7	15400	2-^3A_2 ($1b_2 \rightarrow 2b_1$) $d_{xy} \rightarrow d_{x2-y2}$
8	15460	1329	0.83, 0.83, 1.92	45.7, 45.7, 8.6	13650	2-^3E ($2e \rightarrow 2b_1$) $d_{xz,yz} \rightarrow d_{x2-y2}$
9	17150	1586	0.5, 0.5, 3.75	49.6, 49.6, 0.9	18200,18400	3-^3E ($2e \rightarrow 2a_1$) $d_{xz,yz} \rightarrow d_{z2}$
10	20200	1585	0.91, 0.91, 1.5	42.2, 42.2, 15.6	28900	3-^3A_2 ($1b_2 \rightarrow 2a_1$) $d_{xy} \rightarrow d_{z2}$
11	22830	1500	0.87, 0.87, 1.74	44.5, 44.5, 11		LMCT
12	25700	1782	1.07, 1.07, 0.63	20.7, 20.7, 58.6	31500	$^3[A_1 \oplus A_2 \oplus B_1 \oplus B_2]$

DMM

band	energy (cm^{-1})	line width (cm^{-1})	dipole products Myz, Mxz, Mxy	%polarization (x,y,z)	energy calc. (cm^{-1})	assignment
4	10320	310	0.92, 0.92, 1.91	44.8, 44.8, 10.3	13430	4-^3E ($1b_2 \rightarrow 2e$) $d_{xy} \rightarrow d_{xz,yz}$
5	10910	310	0.92, 0.92, 1.91	44.8, 44.8, 10.3	14000	4-^3E ($1b_2 \rightarrow 2e$) $d_{xy} \rightarrow d_{xz,yz}$
6	13277	1775	0.92, 0.92, 1.95	45.0, 45.0, 10.0	13540	2-^3E ($2e \rightarrow 2b_1$) $d_{xz,yz} \rightarrow d_{x2-y2}$
7	13480	2323	1.39, 1.39, -3.50	46.4, 46.4, 7.3	15400	2-^3A_2 ($1b_2 \rightarrow 2b_1$) $d_{xy} \rightarrow d_{x2-y2}$
8	15450	1994	0.92, 0.92, 1.95	45.0, 45.0, 10.0	13670	2-^3E ($2e \rightarrow 2b_1$) $d_{xz,yz} \rightarrow d_{x2-y2}$
9	17120	2379	0.43, 0.43, 7.67	49.9, 49.9, 0.2	18200,18300	3-^3E ($2e \rightarrow 2a_1$) $d_{xz,yz} \rightarrow d_{z2}$
10	20160	2850	0.66, 0.66, 4.99	49.6, 49.6, 0.9	29400	3-^3A_2 ($1b_2 \rightarrow 2a_1$) $d_{xy} \rightarrow d_{z2}$
11	22830	2850	1.10, 1.10, 1.00	31.2, 31.2, 37.7		LMCT
12	25700	1942	0.98, 0.98, 1.27	38.6, 38.6, 22.7	31300	$^3[A_1 \oplus A_2 \oplus B_1 \oplus B_2]$

N2Q

band	energy (cm^{-1})	line width (cm^{-1})	dipole products Myz, Mxz, Mxy	%polarization (x,y,z)	energy calc. (cm^{-1})	assignment
4	10090	360	0.46, 0.46, 2.69	49.3, 49.3, 1.4	12580	4-^3E ($1b_2 \rightarrow 2e$) $d_{xy} \rightarrow d_{xz,yz}$
5	10120	360	0.46, 0.46, 2.69	49.3, 49.3, 1.4	13610	4-^3E ($1b_2 \rightarrow 2e$) $d_{xy} \rightarrow d_{xz,yz}$
6	11150	1714	0.40, 0.40, 3.09	49.6, 49.6, 0.8	12520	2-^3E ($2e \rightarrow 2b_1$) $d_{xz,yz} \rightarrow d_{x2-y2}$
7	11180	1509	0.99, 0.99, -1.27	38.3, 38.3, 23.4	14020	2-^3A_2 ($1b_2 \rightarrow 2b_1$) $d_{xy} \rightarrow d_{x2-y2}$
8	12860	1449	0.40, 0.40, 3.09	49.6, 49.6, 0.8	13050	2-^3E ($2e \rightarrow 2b_1$) $d_{xz,yz} \rightarrow d_{x2-y2}$
9	14170	1875	0.21, 0.21, 4.50	49.9, 49.9, 0.1	14110,16410	3-^3E ($2e \rightarrow 2a_1$) $d_{xz,yz} \rightarrow d_{z2}$
10	17900	2066	0.79, 0.79, 0.25	8.5, 8.5, 82.9	27100	3-^3A_2 ($1b_2 \rightarrow 2a_1$) $d_{xy} \rightarrow d_{z2}$
11	22440	2325	1.07, 1.07, -1.81	42.6, 42.6, 14.9		LMCT
12	25350	1660	0.64, 0.64, 1.30	44.5, 44.5, 10.9	31000	$^3[A_1 \oplus A_2 \oplus B_1 \oplus B_2]$
13	26990	1050	1.25, 1.25, -0.74	20.4, 20.4, 59.2	34400	$^3[A_1 \oplus A_2 \oplus B_1 \oplus B_2]$

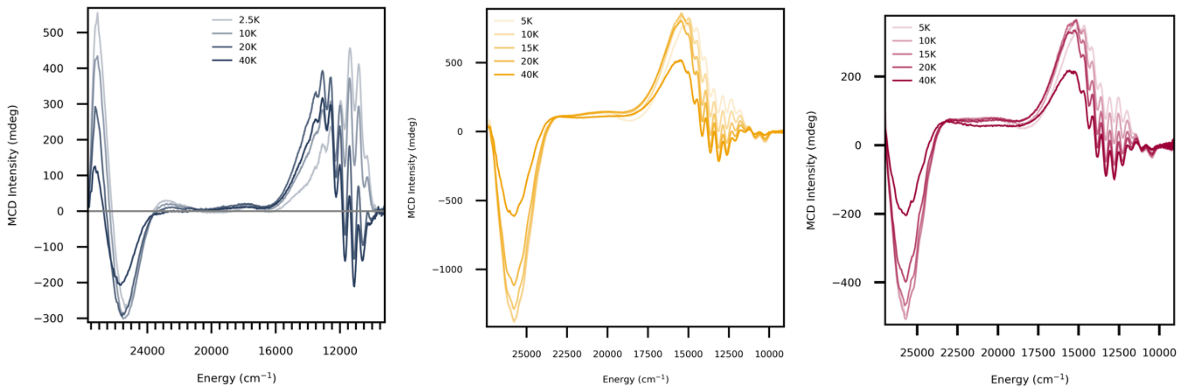

Figure S2. VT 10T MCD for N2Q, DMM, and N4py.

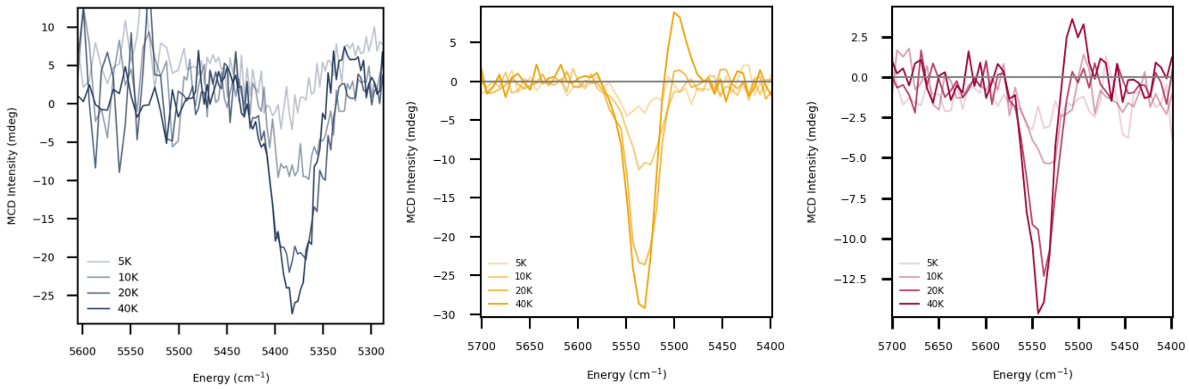

Figure S3. VT 10T MCD of the low-energy NIR feature for N2Q, DMM, and N4py

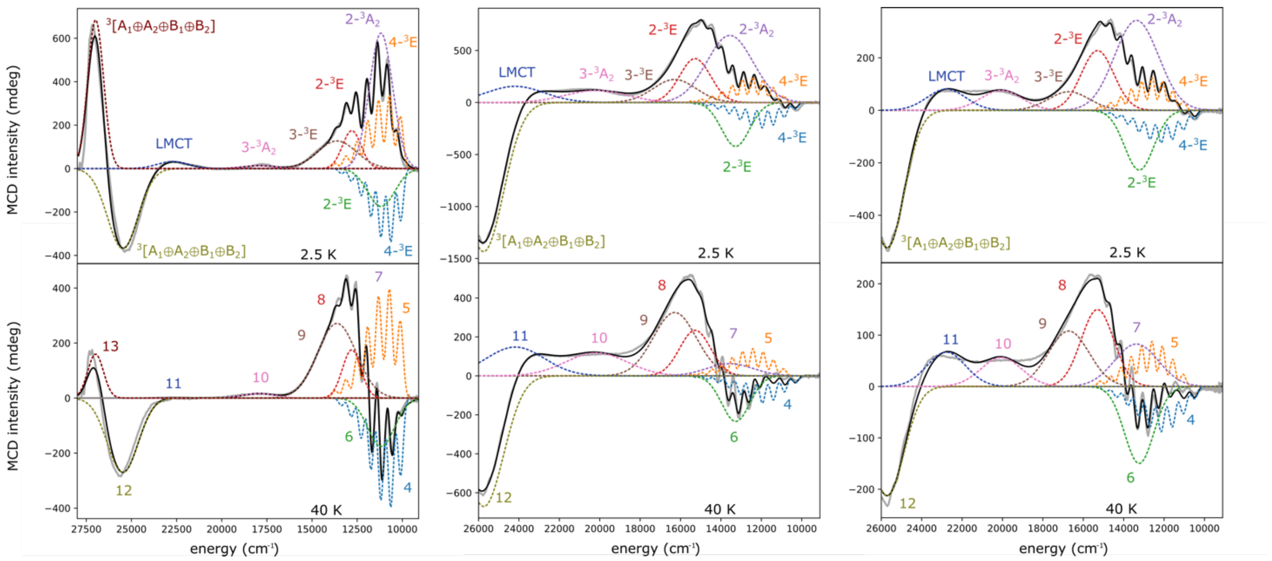

Figure S4. MCD fits for 2.5 and 40K. Spectral fits for the **N2Q** (left), **DMM** (center), **N4py** (right) at 2.5 K (top) and 40 K (bottom). Bottom figures shows band number used in table and fits.

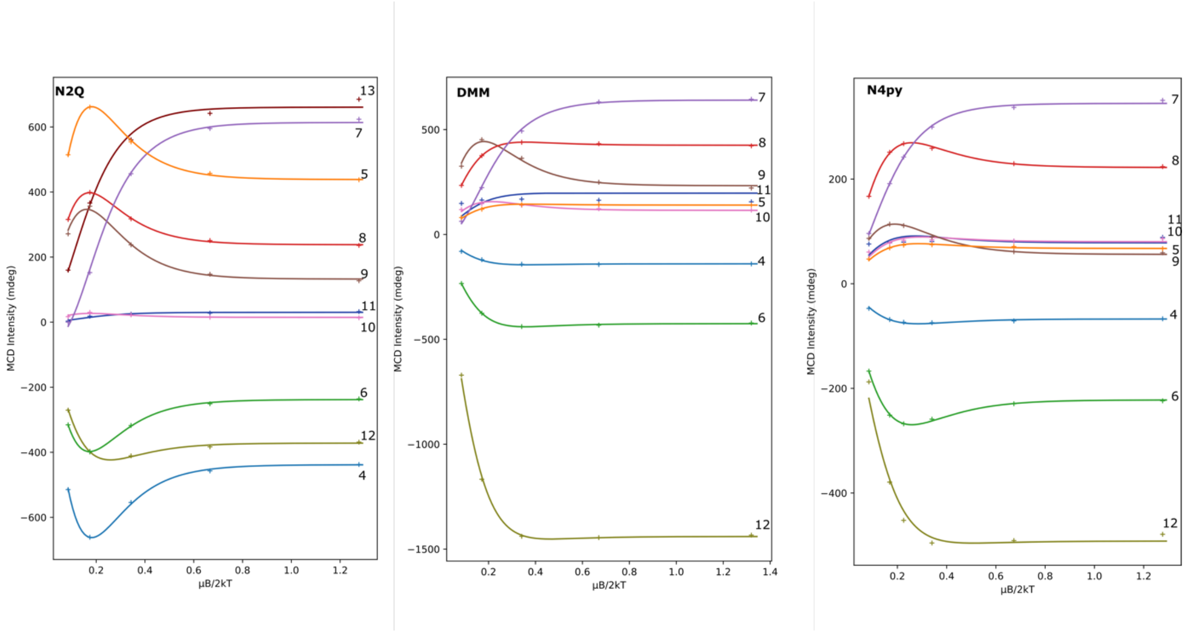

Figure S5. **Spin Hamiltonian simulations.** Spin Hamiltonian simulations for the MCD data and fits in the UV/Vis region between 2.5 and 40 K with 10T applied field for **N2Q**, **DMM**, and **N4py**. Peak numbers are indicated on the right.

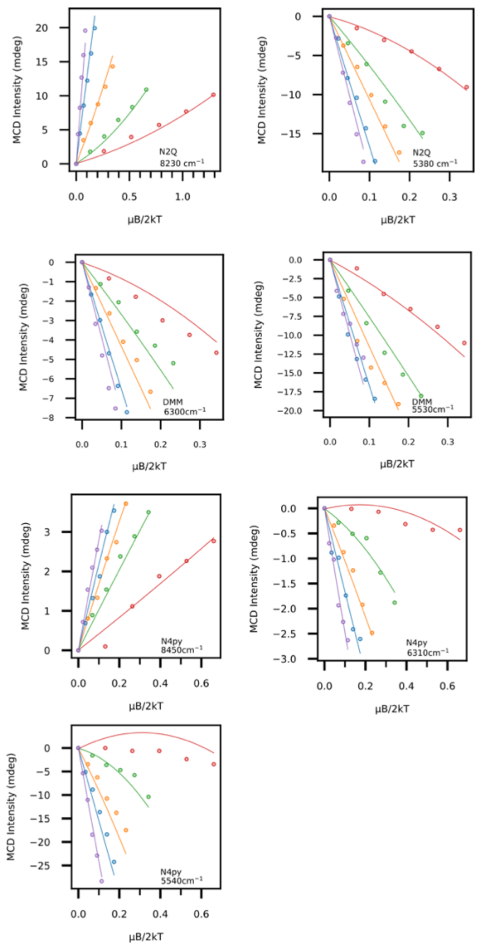

Figure S6. VTVH magnetization curves. VTVH MCD magnetization curves for NIR features in all three complexes with SH simulation. Values used are: **N2Q** D=24.3, E=0, g_z= 2.08, $g_{x,y}$=2.0 **DMM** D = 22.5, E = 0, g_z = 1.95, $g_{x,y}$ = 2.03, **N4py** D = 22.0, E = 0, g_z = 1.95, $g_{x,y}$ = 2.03.

MCD of Glass and Mull sample

The glass and mull powder MCD spectra are overlaid in Figure S7. The singlet transitions are shown to be insensitive to the sample environment.

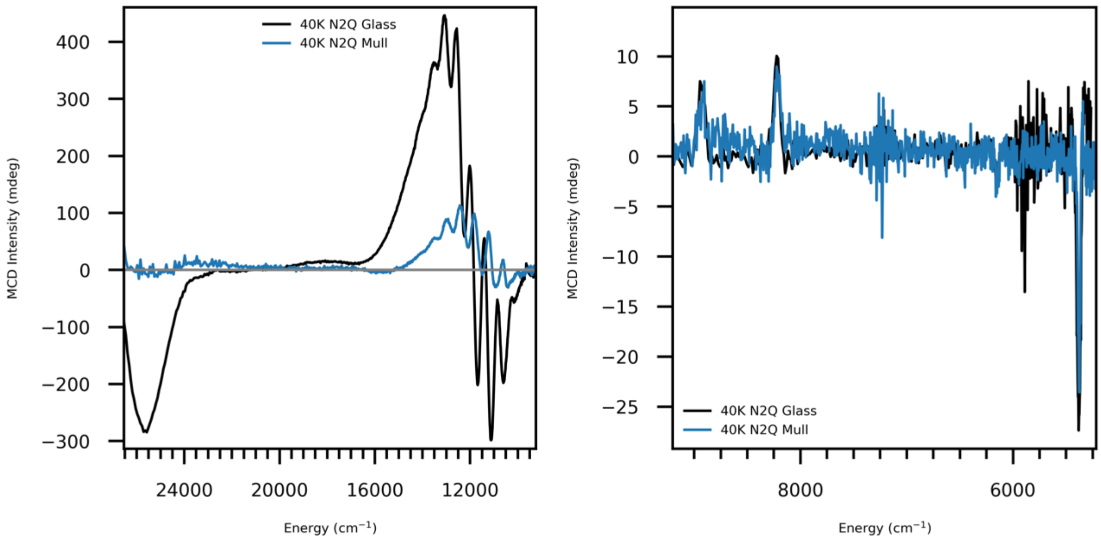

Figure S7. Comparion of MCD at 10T and 40K for N2Q of glass and mull sample. MCD at 10T and 40K for **N2Q** of a frozen solution glass sample and a mull sample over the UV/Vis range (left) and NIR range (right). The low-energy features in the NIR region remain unchanged in energy.

Vibronic Structure.

Bands 1 and 2 have been previously assigned in similar iron(IV)-oxo complexes as the $^3A_2 \rightarrow {}^3E(1b_2(d_{xy}) \rightarrow 2e(d_{xz/yz}))$ transition, so that the spacing between the peaks arise from the excited state Fe—O stretching frequency ($h\nu_{es}$) (*26–28*). Since this is an excitation into the Fe—O π* orbitals, this elongates the bond, reducing the force constant and lowering the vibrational energy of the stretching mode relative to the ground state. The band shape from the intensity of each band is described by a Poisson distribution:

$$\frac{I_{0-n}}{I_{0-0}} = \frac{{S_{HR}}^n}{n!}$$

Where I_{0-0} and I_{0-n} represent the vibronic transitions from the 0 to 0, and the respective 0 to n vibrational energy levels, S_{HR} is the Huang-Rhys factor described by:

$$S_{HR} = \frac{\frac{1}{2}k_{es}\Delta r^2}{h\nu_{es}}$$

Where k_{es} is the excited state force constant, Δr is the distortion in the excited state structure which for this transition represents an elongation in the Fe—O bond, and $h\nu_{es}$ is the excited state vibrational energy. The Huang-Rhys factor represents the change in the potential energy surface due to geometric distortions of the excited state structure. For **N4py**, $h\nu_{es}$ was reported to be ~500 cm^{-1} and S_{HR} was reported to be ~4.5 (*26*). Fits here give values of $h\nu_{es}$ = 530 +/- 18 cm^{-1} and S_{HR} a value of 4.48. For **DMM**, the shape and intensity of the vibronic progression is similar to that of **N4py**, and values of $h\nu_{es}$ = 524 +/- 20 cm^{-1} and S_{HR} = 4.55 were derived from the fits. **N2Q** shows a different band structure with a significantly populated 0—0 band, and with greater individual band intensities overall. The values derived from the fits of **N2Q** are $h\nu_{es}$ = 572 +/- 28 cm^{-1} and S_{HR} = 2.81.

Table S5. Parameters derived from fits of the vibronic structure.

	$h\nu_{es}$ (cm^{-1})	S_{HR}
**N2Q**	572 +/- 28	2.81
**DMM**	524 +/- 20	4.55
**N4py**	530 +/- 18	4.48

Iron(II) RIXS

The RIXS cuts for the iron(II) complexes of **N2Q-2+**, **DMM-2+**, and **N4py-2+** are shown in Figure S9. The high-spin (HS) **N2Q-2+** has a well separated peak at 1.2 eV at all incident energies with a shoulder that grows in at higher incident energies. This peak corresponds to the spin-allowed dd transitions (Figure S8 and FigureS9). At higher energy transfer there are overlapping features around 2.6 eV that grow in relative intensity with increasing incident energy, and with 710.6 eV incident energy a third feature around 4 eV energy transfer. The low-spin (LS) **DMM-2+** and **N4py-2+** are very similar, with significantly more overlapping features than the HS iron(II) complex. In the 708.1 and 708.6 eV cuts, there are peaks at 1.5 and 2.0 eV that lose definition in the higher incident energy 710.6 eV cut.

The HS iron(II) complex, **N2Q-2+**, is calculated to have a nearly degenerate S = 2 ground state, and a low-lying S = 2 state 0.124 eV above the ground state. The next lowest lying states are S = 2 at 1.3 eV, with minimal splitting implying nearly degenerate e_g orbitals. At higher energies there are two sets of densely packed spin-forbidden transitions, which align with the shoulder at 1.5 eV that gains intensity at higher incident energy, and the second, higher energy peak at 2.6 eV.

The LS iron(II) complexes, **N4py-2+** and **DMM-2+**, are both calculated to have no states lower in energy than approximately 1.5 eV. There are a multitude of ΔS = +1/+2 transitions between 1.5 and 2.5 eV, where at 2.5 eV the first ΔS = 0 transitions are calculated to be. The ΔS = 1 transitions line up nicely with the features at approximately 1.5 and 2.0 eV, and the first ΔS = 0 transition is in agreement with the large peak at approximately 2.3 eV in the 708.1 and 710.6 eV cuts. The largest peak shifts to 2.5 eV in the 708.6 eV cut, though the CAS calculations are underestimating this transition by ~0.1 eV.

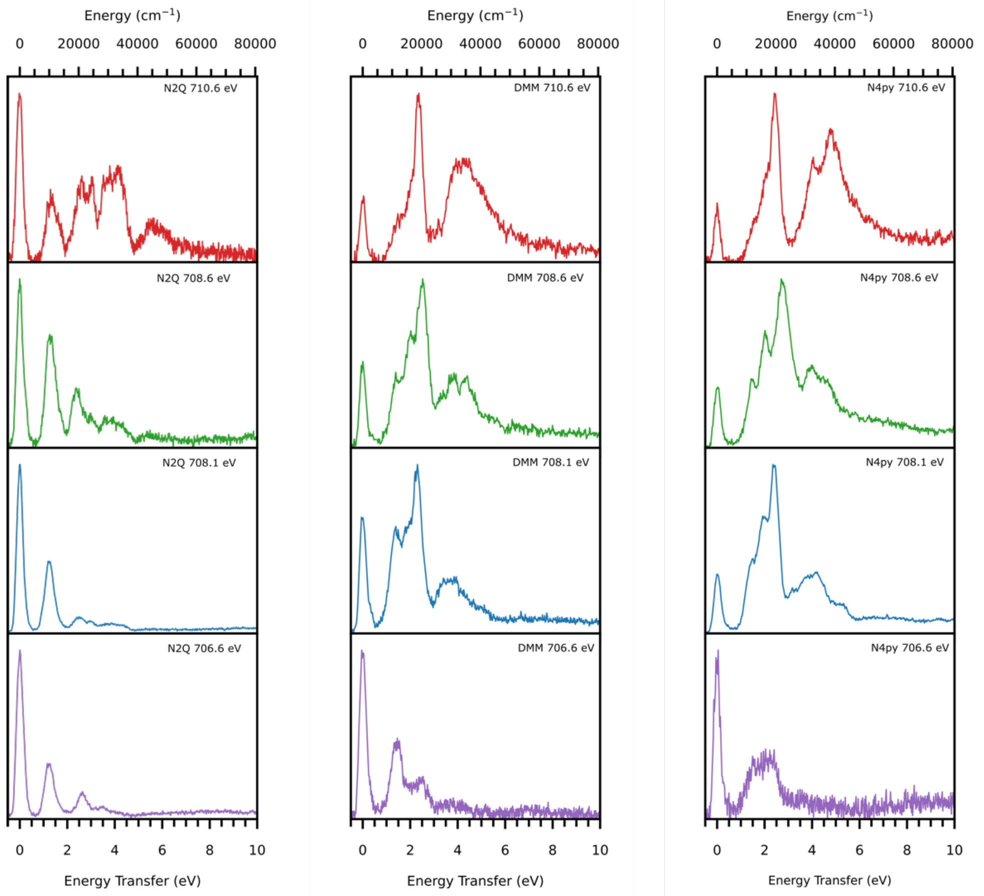

Figure S8. RIXS cuts for iron(II) complexes. RIXS cuts for the iron(II) complexes of **N2Q**, **DMM**, and **N4py** out to 10 eV energy transfer. Incident energies are shown in figure for each cut.

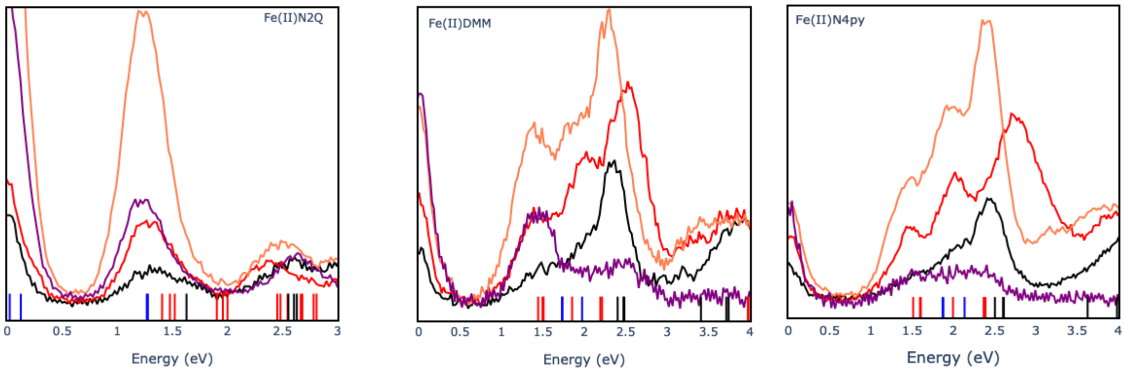

Figure S9. **RIXS cuts for iron(II) complexes with CASSCF/NEVPT2 energies.** RIXS cuts for iron(II) complexes and their state-averaged CASSCF/NEVPT2 energies represented below as sticks with spin states S=0 (black), S=1 (red) and S=2 (blue). The ground states are **N2Q** (S=2), **DMM** (S=0), and **N4py** (S=0).

Iron(IV)-oxo RIXS

The 2p3d RIXS for the three complexes are shown in Figure S10. The cut with a 706.6 eV incident energy displays two separated features for all three complexes, with the sharper, more intense peak at approximately at 1.3 eV and the broader, weaker feature at approximately 2.6 eV. Increasing the incident energy to 708.1 eV causes a loss of definition in the complexes, but in **N4py** and **DMM** a feature grows in at around 2.2 eV, while in **N2Q** the intensity peaks around 1.5 eV. With an incident energy of 708.6 eV, **N4py** remains largely unchanged while **DMM** and **N2Q** becomes more well-defined. Further increasing the incident energy to 710.6 eV causes significant changes in the RIXS, with intensity below 1 eV becoming apparent in all complexes, as well as a shoulder around 1.4 eV and a peak around 2.3 eV. For the lower energy transfer feature, in **N4py** is appears as a small shoulder around 1 eV, the **DMM** shows a larger separation and is around 0.85 eV, and the **N2Q** appears as a distinct peak at approximately 0.7 eV. Fits of the data are performed both with energies held (Figure S11) at the values from the MCD fits, as well as using freely floating peak energies (Figure S12). In the 0.4 to 1.0 eV range, both fits contain a constant Gaussian function at approximately 0.66 eV, which in the MCD-derived fits corresponds to the lowest triplet to singlet transition. From the MCD fitting, the **N2Q**, **DMM** and **N4py** have a function at 0.45 eV, 0.86 and 0.94 eV, respectively. From the freely floating peak fitting, the features are at 0.47, 0.85, and 0.95 for **N2Q**, **DMM**, and **N4py**, respectively. As the triplet to singlet transitions remain at nearly constant energy for all three complexes, the change in shape of the 708.6 and 710.6 eV cut requires a Gaussian function that shifts significantly in energy between complexes. This is consistent in both methods of fitting the RIXS cuts. In Figure S13, the fits are shown excluding the $^5A_1$ peak, and the reduced χ^2 are shown in table S7.

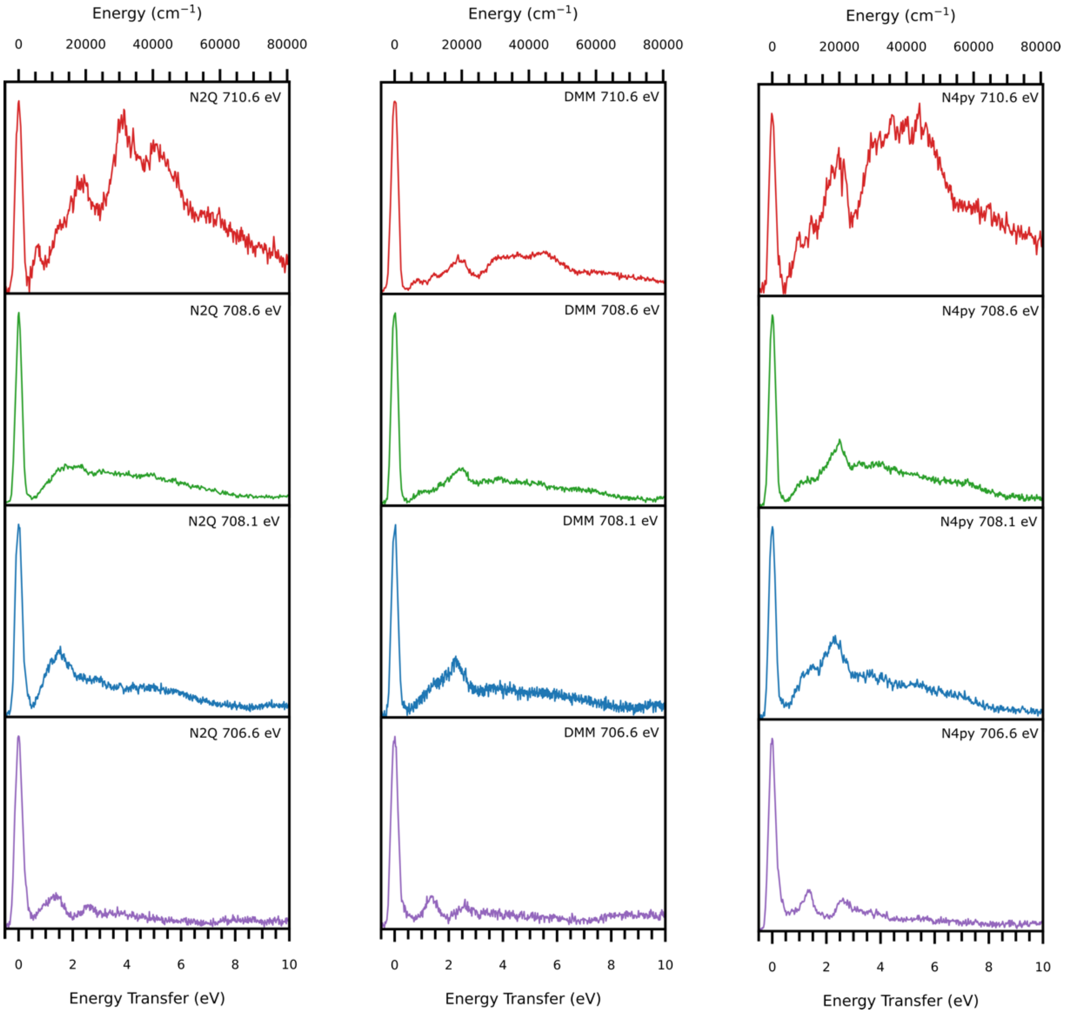

Figure S10. **RIXS cuts for iron(IV)-oxo complexes.** RIXS cuts for the iron(IV)-oxo complexes of **N2Q**, **DMM**, and **N4py**

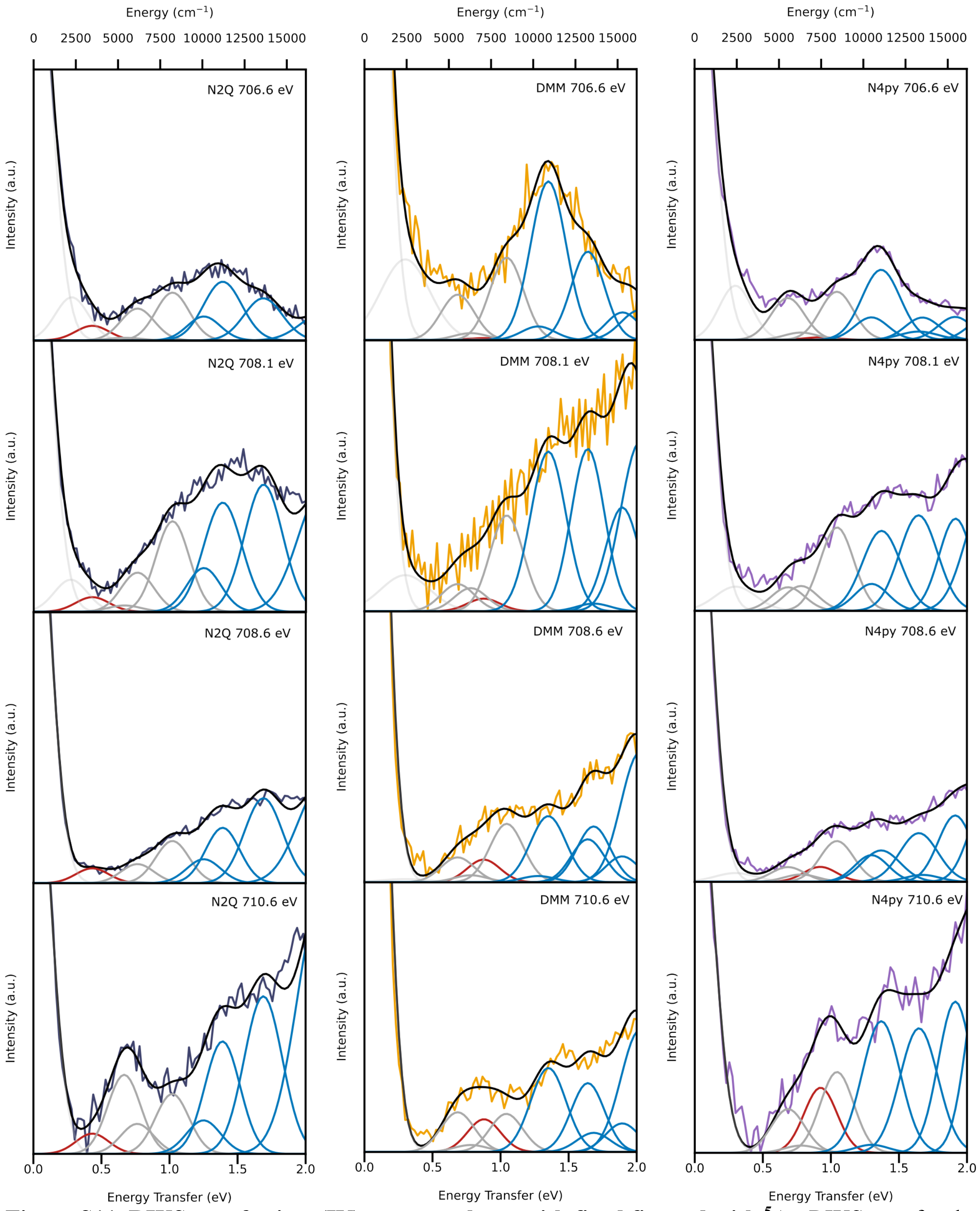

Figure S11. RIXS cuts for iron(IV)-oxo complexes with fixed fits and with $^5A_1$. RIXS cuts for the iron(IV)-oxo complexes with fitted functions with energies fixed for the singlet and triplet transitions from the MCD fits. Linewidths were allowed to float (see methods). The red (quintet) function's energy is freely floating, and found to be 0.45 (**N2Q**), 0.86 (**DMM**), and 0.94 (**N4py**) eV. A single function (light grey) is used to fit asymmetry in the elastic peak in certain incident energies.

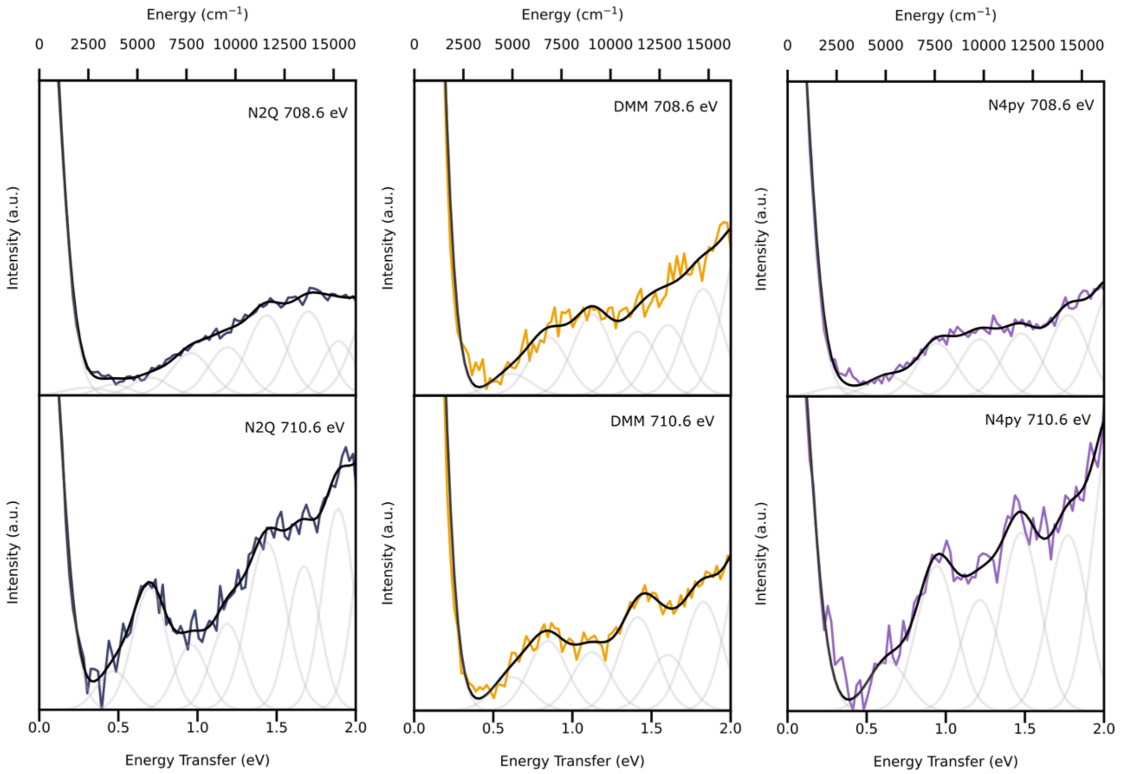

Figure S12. RIXS cuts for iron(IV)-oxo complexes with floated fits. RIXS cuts for the iron(IV)-oxo complexes with fitted functions with energies freely floating. Below 1 eV, all three complexes have a fitted function at ~0.66 eV corresponding to the singlet transition, and a moving function from 0.47 (**N2Q**), 0.85 (**DMM**), and 0.95 (**N4py**) corresponding to the $^5A_1$ feature.

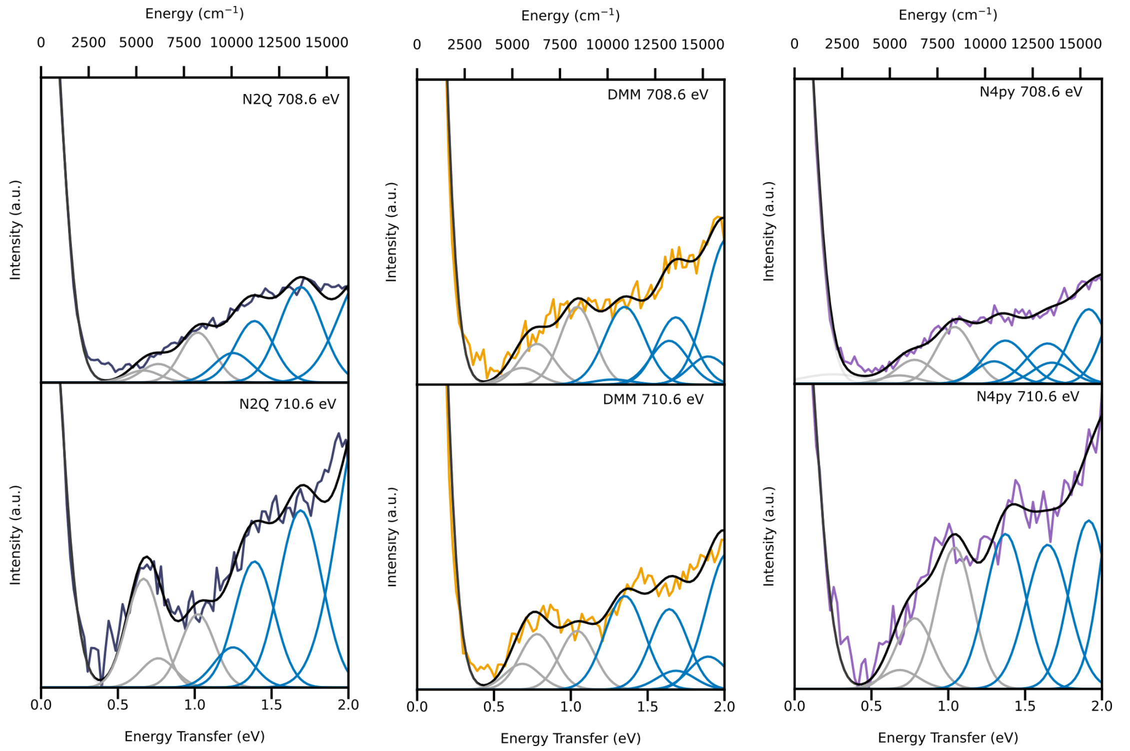

Figure S13. RIXS cuts for the iron(IV)-oxo complexes with MCD held intensities without the floated peak for the $^5A_1$ state

Table S6. Reduced χ^2 for the fits with and without the $^5A_1$ function for the RIXS cuts at 708.6 and 710.6 eV.

Complex	Incident Energy (eV)	With $^5A_1$	Without $^5A_1$
**N2Q**	708.6	2.2	3.3
	710.6	9.4	13.8
**DMM**	708.6	4.5	4.9
	710.6	5.9	6.2
**N4py**	708.6	2.2	3.4
	710.6	13.5	17.1

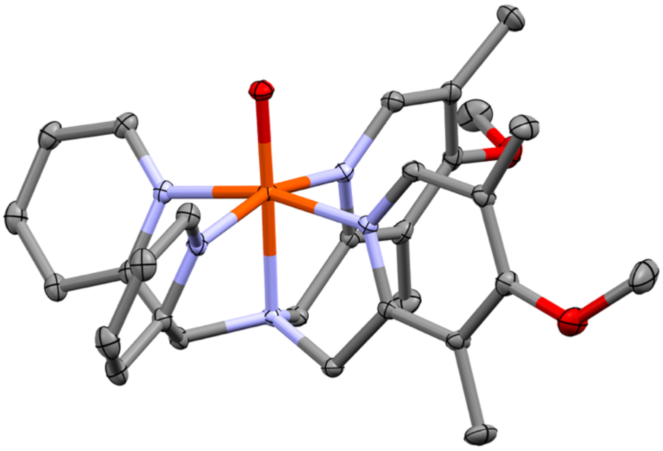

Figure S14. ORTEP of DMM. ORTEP plots of **DMM** with thermal ellipsoids set at 50% probability. Hydrogens and counterions are removed for clarity.

Table S7. Selected geometric parameters for the iron(IV)-oxo complexes

	[a]**N4py**	[b]**DMM**	[c]**N2Q**
**Fe-O**	1.639	1.659	1.677
**Fe-N$_{eq,py}$**	1.964	1.969<br>1.968	2.023<br>2.024
**Fe-N$_{eq,het}$**	1.949	1.968<br>1.946	2.073<br>2.067
**Fe-N$_{ax}$**	2.033	2.047	2.084
**N$_{ax}$-Fe-O**	179.4	178.1	170.5

[a] Data from reference *19*
[b] Data from this work
[c] Data from reference (*21*)

Table S8. Crystallographic data of **DMM**.

	**DMM**
Formula	$C_{29}H_{33}Cl_2FeN_5O_{11}$
M_r in g mol^{-1}	754.35
Color, habit	blue prism
Crystal system	tetragonal
Space group	$I4_1/a$; No. 88
a in Å	34.1866(12)
b in Å	34.1866(12)
c in Å	10.6065(4)
V in Å^3	12396.1(10)
Z	16
T in K	100(2)
Crystal size in mm^3	0.11 × 0.085 × 0.082
ρ_c in g cm^{-3}	1.617
F(000)	6240
Diffractometer	Bruker-AXS Kappa Mach3 APEX-II
$\lambda_{XK\alpha}$ in Å	X = Mo<br>0.71073
θ_{min} in °<br>θ_{max} in °	1.191<br>31.221
Index range	$-49 \leq h \leq 49$<br>$-49 \leq k \leq 49$<br>$-15 \leq l \leq 15$
μ in mm^{-1}	0.730
Abs. correction	Multi-Scan
Reflections collected	207278
Reflections unique	10063
R_{int}	0.0329
Reflections obs. [F>2σ(F)]	8785
Residual density in e Å^{-3}	0.558/-0.596
Params/restraints	439/0
GOOF	1.052
R_1 [I>2σ(I)]	0.0337
wR_2 (all data)	0.0875
CCDC	2314930

Mössbauer for iron(IV)-oxo complexes

The 2K, zero-field Mössbauer spectra are shown in figure S16 for the three oxo complexes. As the solid powders could be generated in large quantities, unenriched samples were used for these measurements. The fitted parameters are shown in Table S8. As only one quadrupole doublet was necessary in simulation for the fits, the samples are composed purely of the S=1 iron(IV)-oxo complexes within the limit of detection of the method.

Table S9. Mössbauer parameters

Complex	δ (mm s^{-1})	$\lvert\Delta E_Q\rvert$ (mm s^{-1})
**N2Q**	0.04	0.64
**DMM**	-0.03	0.94
**N4py**	-0.04	0.91

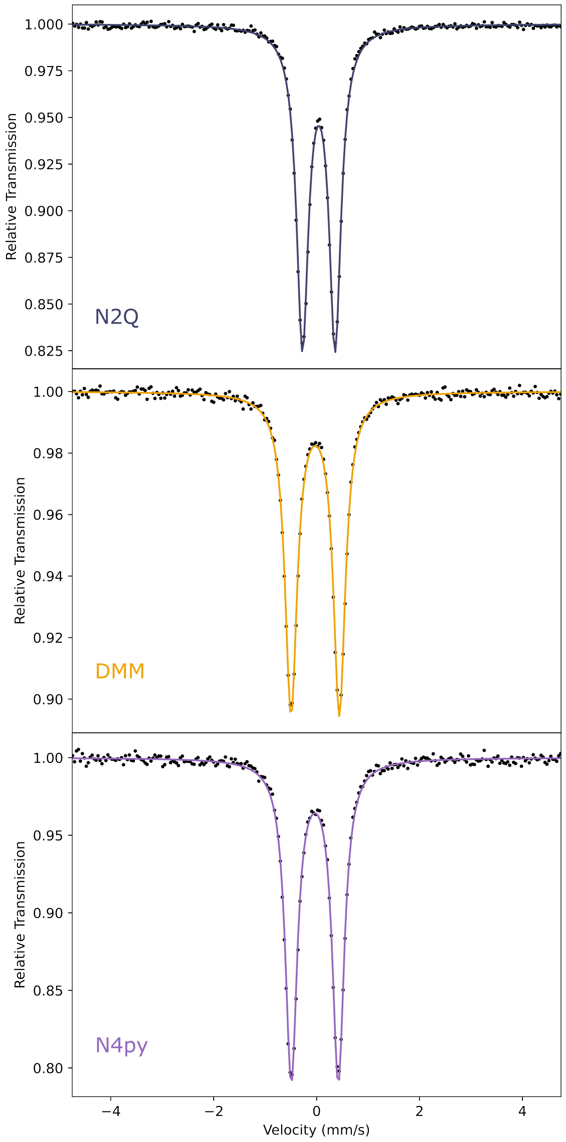

Figure S15. Mössbauer spectra for iron(IV)-oxo complexes. 2K, 0T Mössbauer spectra of **N2Q** (top), **DMM** (middle), and **N4py** (bottom). All complexes are simulated a single quadrupole doublet with parameters given in Table S9.

REFERENCES AND NOTES

1. D. Schröder, S. Shaik, H. Schwarz, Ttwo-state reactivity as a new concept in organometallic chemistry. *Acc. Chem. Res.* **33**, 139–145 (2000).

2. S. Shaik, D. Danovich, A. Fiedler, D. Schröder, H. Schwarz, Two-state reactivity in organometallic gas-phase ion chemistry. *Helv. Chim. Acta* **78**, 1393–1407 (1995).

3. L. Pauling, C. D. Coryell, The magnetic properties and structure of hemoglobin, oxyhemoglobin and carbonmonoxyhemoglobin. *Proc. Natl. Acad. Sci. U.S.A.* **22**, 210–216 (1936).

4. D. Danovich, S. Shaik, Spin−orbit coupling in the oxidative activation of H−H by FeO+. Selection rules and reactivity effects. *J. Am. Chem. Soc.* **119**, 1773–1786 (1997).

5. J. C. Price, E. W. Barr, B. Tirupati, J. M. Bollinger, C. Krebs, The first direct characterization of a high-valent iron intermediate in the reaction of an alpha-ketoglutarate-dependent dioxygenase: a high-spin FeIV complex in taurine/alpha-ketoglutarate dioxygenase (TauD) from Escherichia coli. *Biochemistry* **42**, 7497–508 (2003).

6. J.-U. Rohde, J.-H. In, M. H. Lim, W. W. Brennessel, M. R. Bukowski, A. Stubna, E. Münck, W. Nam, L. Que, Crystallographic and spectroscopic characterization of a nonheme Fe(IV)=O complex. *Science* **299**, 1037–1039 (2003).

7. M. Puri, L. Que, Toward the synthesis of more reactive S = 2 non-heme oxoiron(IV) complexes. *Acc. Chem. Res.* **48**, 2443–52 (2015).

8. S. Sinnecker, N. Svensen, E. W. Barr, S. Ye, J. M. Bollinger, F. Neese, C. Krebs, Spectroscopic and computational evaluation of the structure of the high-spin Fe(IV)-Oxo intermediates in Taurine: α-Ketoglutarate dioxygenase from Escherichia coli and its His99Ala ligand variant. *J. Am. Chem. Soc.* **129**, 6168–6179 (2007).

9. J. Hohenberger, K. Ray, K. Meyer, The biology and chemistry of high-valent iron–oxo and iron–nitrido complexes. *Nat. Commun.* **3**, 720 (2012).

10. A. Nandy, H. J. Kulik, Why conventional design rules for C–H activation fail for open-shell Transition-metal catalysts. *ACS Catal.* **10**, 15033–15047 (2020).

11. D. Mandal, D. Mallick, S. Shaik, Kinetic isotope effect determination probes the spin of the transition state, its stereochemistry, and its ligand sphere in hydrogen abstraction Reactions of Oxoiron(IV) complexes. *Acc. Chem. Res.* **51**, 107–117 (2018).

12. S. Shaik, Two-state reactivity: Personal recounting of its conception and future prospects. *Israel J. Chem.* **60**, 938–956 (2020).

13. K. B. Cho, H. Chen, D. Janardanan, S. P. de Visser, S. Shaik, W. Nam, Nonheme iron-oxo and -superoxo reactivities: O2 binding and spin inversion probability matter. *Chem. Commun.* **48**, 2189–91 (2012).

14. D. Janardanan, Y. Wang, P. Schyman, L. Que, S. Shaik, The fundamental role of exchange-enhanced reactivity in C-H activation by S=2 oxo iron(IV) complexes. *Angew. Chem. Int. Ed.* **49**, 3342–5 (2010).

15. H. Hirao, D. Kumar, L. Que, S. Shaik, Two-state reactivity in alkane hydroxylation by non-heme iron-oxo complexes. *J. Am. Chem. Soc.* **128**, 8590–606 (2006).

16. S. Shaik, H. Chen, D. Janardanan, Exchange-enhanced reactivity in bond activation by metal–oxo enzymes and synthetic reagents. *Nat. Chem.* **3**, 19–27 (2011).

17. S. Ye, C. Y. Geng, S. Shaik, F. Neese, Electronic structure analysis of multistate reactivity in transition metal catalyzed reactions: the case of C-H bond activation by non-heme iron(IV)-oxo cores. *Phys. Chem. Chem. Phys.* **15**, 8017–30 (2013).

18. C. Geng, S. Ye, F. Neese, Analysis of reaction channels for alkane hydroxylation by nonheme iron(IV)–Oxo Complexes. *Angew. Chem. Int. Ed.* **49**, 5717–5720 (2010).

19. E. Andris, J. Jašík, L. Gómez, M. Costas, J. Roithová, Innentitelbild: Spectroscopic characterization and reactivity of triplet and Quintet Iron(IV) Oxo complexes in the gas phase. *Angew. Chem. Int. Ed.* **128**, 3578–3578 (2016).

20. E. Andris, R. Navratil, J. Jasik, T. Terencio, M. Srnec, M. Costas, J. Roithova, Chasing the evasive Fe═O stretch and the spin state of the iron(IV)-Oxo complexes by photodissociation spectroscopy. *J. Am. Chem. Soc.* **139**, 2757–2765 (2017).

21. W. Rasheed, A. Draksharapu, S. Banerjee, V. G. Young, R. Fan, Y. Guo, M. Ozerov, J. Nehrkorn, J. Krzystek, J. Telser, L. Que, Crystallographic evidence for a sterically induced ferryl tilt in a non-heme Oxoiron(IV) complex that makes it a better oxidant. *Angew. Chem. Int. Ed.* **57**, 9387–9391 (2018).

22. J. O. Bigelow, J. England, J. E. Klein, E. R. Farquhar, J. R. Frisch, M. Martinho, D. Mandal, E. Munck, S. Shaik, L. Que, Oxoiron(IV) tetramethylcyclam complexes with axial carboxylate ligands: Effect of tethering the carboxylate on reactivity. *Inorg. Chem.* **56**, 3287–3301 (2017).

23. B. M. Flöser, Y. Guo, C. Riplinger, F. Tuczek, F. Neese, Detailed pair natural orbital-based coupled cluster studies of spin crossover energetics. *J. Chem. Theory Comput.* **16**, 2224–2235 (2020).

24. Q. M. Phung, C. Martin-Fernandez, J. N. Harvey, M. Feldt, Ab Initio calculations for spin-gaps of non-heme iron complexes. *J. Chem. Theory Comput.* **15**, 4297–4304 (2019).

25. M. Drosou, C. A. Mitsopoulou, D. A. Pantazis, Reconciling local coupled cluster with multireference approaches for transition metal spin-state energetics. *J. Chem. Theory Comput.* **18**, 3538–3548 (2022).

26. A. Decker, J. U. Rohde, E. J. Klinker, S. D. Wong, L. Que, E. I. Solomon, Spectroscopic and quantum chemical studies on low-spin FeIV=O complexes: Fe-O bonding and its contributions to reactivity. *J. Am. Chem. Soc.* **129**, 15983–96 (2007).

27. S. Ye, C. Kupper, S. Meyer, E. Andris, R. Navratil, O. Krahe, B. Mondal, M. Atanasov, E. Bill, J. Roithova, F. Meyer, F. Neese, Magnetic circular dichroism evidence for an unusual electronic structure of a tetracarbene-Oxoiron(IV) complex. *J. Am. Chem. Soc.* **138**, 14312–14325 (2016).

28. A. Decker, J. U. Rohde, L. Que, E. I. Solomon, Spectroscopic and quantum chemical characterization of the electronic structure and bonding in a non-heme FeIV[double bond]O complex. *J. Am. Chem. Soc.* **126**, 5378–9 (2004).

29. C. Kupper, B. Mondal, J. Serrano-Plana, I. Klawitter, F. Neese, M. Costas, S. Ye, F. Meyer, Nonclassical single-state reactivity of an Oxo-Iron(IV) complex confined to triplet pathways. *J. Am. Chem. Soc.* **139**, 8939–8949 (2017).

30. H. Lu, M. Rossi, A. Nag, M. Osada, D. F. Li, K. Lee, B. Y. Wang, M. Garcia-Fernandez, S. Agrestini, Z. X. Shen, E. M. Been, B. Moritz, T. P. Devereaux, J. Zaanen, H. Y. Hwang, K.-J. Zhou, W. S. Lee, Magnetic excitations in infinite-layer nickelates. *Science* **373**, 213–216 (2021).

31. A. Nag, H. C. Robarts, F. Wenzel, J. Li, H. Elnaggar, R. P. Wang, A. C. Walters, M. Garcia-Fernandez, F. M. F. de Groot, M. W. Haverkort, K. J. Zhou, Many-body physics of single and double spin-flip excitations in NiO. *Phys. Rev. Lett.* **124**, 067202 (2020).

32. M. Al Samarai, A. W. Hahn, A. Beheshti Askari, Y. T. Cui, K. Yamazoe, J. Miyawaki, Y. Harada, O. Rudiger, S. DeBeer, Elucidation of structure-activity correlations in a nickel manganese oxide oxygen evolution reaction catalyst by operando Ni L-Edge x-ray absorption spectroscopy and 2p3d resonant inelastic x-ray scattering. *ACS Appl. Mater. Interfaces* **11**, 38595–38605 (2019).

33. X. Ding, C. C. Tam, X. Sui, Y. Zhao, M. Xu, J. Choi, H. Leng, J. Zhang, M. Wu, H. Xiao, X. Zu, M. Garcia-Fernandez, S. Agrestini, X. Wu, Q. Wang, P. Gao, S. Li, B. Huang, K.-J. Zhou, L. Qiao, Critical role of hydrogen for superconductivity in nickelates. *Nature* **615**, 50–55 (2023).

34. A. W. Hahn, B. E. Van Kuiken, V. G. Chilkuri, N. Levin, E. Bill, T. Weyhermüller, A. Nicolaou, J. Miyawaki, Y. Harada, S. DeBeer, Probing the valence electronic structure of low-spin ferrous and ferric Complexes using 2p3d resonant inelastic x-ray scattering (RIXS). *Inorg. Chem.* **57**, 9515–9530 (2018).

35. A. W. Hahn, B. E. Van Kuiken, M. Al Samarai, M. Atanasov, T. Weyhermüller, Y.-T. Cui, J. Miyawaki, Y. Harada, A. Nicolaou, S. DeBeer, Measurement of the ligand field spectra of ferrous and ferric Iron chlorides using 2p3d RIXS. *Inorg. Chem.* **56**, 8203–8211 (2017).

36. B. E. Van Kuiken, A. W. Hahn, B. Nayyar, C. E. Schiewer, S. C. Lee, F. Meyer, T. Weyhermuller, A. Nicolaou, Y. T. Cui, J. Miyawaki, Y. Harada, S. DeBeer, Electronic spectra of iron-sulfur complexes measured by 2p3d RIXS spectroscopy. *Inorg. Chem.* **57**, 7355–7361 (2018).

37. K. Kunnus, L. Li, C. J. Titus, S. J. Lee, M. E. Reinhard, S. Koroidov, K. S. Kjaer, K. Hong, K. Ledbetter, W. B. Doriese, G. C. O'Neil, D. S. Swetz, J. N. Ullom, D. Li, K. Irwin, D. Nordlund, A. A. Cordones, K. J. Gaffney, Chemical control of competing electron transfer pathways in iron tetracyano-polypyridyl photosensitizers. *Chem. Sci.* **11**, 4360–4373 (2020).

38. K. Kunnus, M. Guo, E. Biasin, C. B. Larsen, C. J. Titus, S. J. Lee, D. Nordlund, A. A. Cordones, J. Uhlig, K. J. Gaffney, Quantifying the steric effect on metal-ligand bonding in Fe carbene photosensitizers with Fe 2p3d resonant inelastic x-ray scattering. *Inorg. Chem.* **61**, 1961–1972 (2022).

39. S. Rana, A. Dey, D. Maiti, Mechanistic elucidation of C-H oxidation by electron rich non-heme iron(IV)-oxo at room temperature. *Chem. Commun.* **51**, 14469–72 (2015).

40. E. J. Klinker, J. Kaizer, W. W. Brennessel, N. L. Woodrum, C. J. Cramer, L. Que, Structures of nonheme oxoiron(IV) complexes from x-ray crystallography, NMR spectroscopy, and DFT calculations. *Angew. Chem. Int. Ed.* **44**, 3690–4 (2005).

41. G. Santiago, M. Kasha, Intraconfigurational spin-forbidden transitions in chromium(III), manganese(II), and nickel(II) complexes in aqueous glass solutions at 77.deg.K. *J. Am. Chem. Soc.* **91**, 757–758 (1969).

42. B. De Souza, F. Neese, R. Izsák, On the theoretical prediction of fluorescence rates from first principles using the path integral approach. *J. Chem. Phys.* **148**, 034104 (2018).

43. F. M. F. de Groot, P. Kuiper, G. A. Sawatzky, Local spin-flip spectral distribution obtained by resonant x-ray raman scattering. *Phys. Rev. B* **57**, 14584–14587 (1998).

44. M. Lubben, A. Meetsma, E. C. Wilkinson, B. Feringa, L. Que Jr., Nonheme iron centers in oxygen activation: Characterization of an iron(III) hydroperoxide intermediate. *Angew. Chemie Int. Ed.* **34**, 1512–1514 (1995).

45. A. A. Massie, M. C. Denler, L. T. Cardoso, A. N. Walker, M. K. Hossain, V. W. Day, E. Nordlander, T. A. Jackson, Equatorial ligand perturbations influence the reactivity of manganese(IV)-Oxo complexes. *Angew. Chem. Int. Ed.* **56**, 4178–4182 (2017).

46. L. Krause, R. Herbst-Irmer, G. M. Sheldrick, D. Stalke, Comparison of silver and molybdenum microfocus x-ray sources for single-crystal structure determination. *J. Appl. Cryst.* **48**, 3–10 (2015).

47. APEX3 v2019.11-0 program package (Bruker AXS, 2019.

48. C. Schulz, K. Lieutenant, J. Xiao, T. Hofmann, D. Wong, K. Habicht, Characterization of the soft x-ray spectrometer PEAXIS at BESSY II. *J. Synchrotron. Rad.* **27**, 238–249 (2020).

49. F. Neese, Software update: The ORCA program system—Version 5.0. *WIREs Computat. Mol. Sci.* **12**, e1606 (2022).

50. K. Ruedenberg, L. M. Cheung, S. T. Elbert, MCSCF optimization through combined use of natural orbitals and the brillouin–levy–berthier theorem. *Int. J. Quantum Chem.* **16**, 1069–1101 (1979).

51. B. O. Roos, P. R. Taylor, P. E. M. Siegbahn, A complete active space SCF method (CASSCF) using a density matrix formulated super-CI approach. *Chem. Phys.* **48**, 157–173 (1980).

52. C. Angeli, R. Cimiraglia, S. Evangelisti, T. Leininger, J. P. Malrieu. Introduction ofn-electron valence states for multireference perturbation theory. *J. Chem. Phys.* **114**, 10252–10264 (2001).

53. A. D. Becke, Density-functional exchange-energy approximation with correct asymptotic behavior. *Phys. Rev. A Gen. Phys.* **38**, 3098 (1988).

54. F. Weigend, R. Ahlrichs, Balanced basis sets of split valence, triple zeta valence and quadruple zeta valence quality for H to Rn: Design and assessment of accuracy. *Phys. Chem. Chem. Phys.* **7**, 3297–305 (2005).

55. G. L. Stoychev, A. A. Auer, F. Neese, Automatic generation of auxiliary basis sets. *J. Chem. Theory Comput.* **13**, 554–562 (2017).

56. S. Ye, G. Xue, I. Krivokapic, T. Petrenko, E. Bill, L. Que, F. Neese, Magnetic circular dichroism and computational study of mononuclear and dinuclear iron(IV) complexes. *Chem. Sci.* **6**, 2909–2921 (2015).
